# Proteomic analysis of equine amniotic mesenchymal stromal cells and their extracellular vesicles: comparing their regenerative properties

**DOI:** 10.20517/evcna.2025.66

**Published:** 2026-03-09

**Authors:** Giulia Gaspari, Alessio Soggiu, Paola Gagni, Pietro Riccaboni, Andrea Cappelleri, Fausto Cremonesi, Anna Lange-Consiglio

**Affiliations:** ^1^Laboratory of Reproduction and Regenerative Medicine, Department of Veterinary Medicine and Animal Science (DIVAS), Università degli Studi di Milano, Lodi 26900, Italy.; ^2^Dipartimento di Scienze Biomediche, Chirurgiche e Odontoiatriche, Università degli Studi di Milano, Milan 20133, Italy.; ^3^Istituto di Scienze e Tecnologie Chimiche “Giulio Natta” (SCITEC), Consiglio Nazionale delle Ricerche (CNR), Milan 20133, Italy.; ^#^Authors contributed equally.

**Keywords:** Amniotic mesenchymal cells, extracellular vesicles, proteomics

## Abstract

**Aim:** To compare specific functional features of equine amniotic mesenchymal cells (eAMCs) and their extracellular vesicles (EVs) through proteomic analysis.

**Methods:** eAMCs were obtained by enzymatic digestion and their EVs were isolated by ultracentrifugation. Cells and EVs were characterized according to ISCT and MISEV guidelines. A proteomic analysis of both eAMCs and EVs was conducted. The raw data files were analyzed using FragPipe 22 and uniprotkb equus_caballus_reviewed database (20.02.2025) to obtain protein identifications (false discovery rate = 0.01) and their respective label-free quantification values using recommended parameters. Statistical analysis was performed based on the combined_protein.tsv file using FragPipe-Analyst. A cutoff of the adjusted *P*-value of 0.05 along with a |log2 fold change| of 1 has been applied to determine differentially expressed proteins in the comparison.

**Results:** A total of 3,631 proteins were identified, of which 3,147 were identified with more than two peptides. Among these, 2,235 were exclusive to eAMCs, 697 were shared between eAMCs and EVs, and 71 were exclusive to EVs. eAMCs and EVs revealed distinguished proteomic profiles, differentially expressing proteins involved in biological processes related to tissue regeneration. Proteins promoting anti-inflammatory activity, oxidative stress resistance and angiogenesis exhibited increased expression levels in eAMCs, while extracellular matrix organization and deposition were predominantly upregulated in EVs.

**Conclusions:** Equine eAMCs and EVs are characterized by a distinct proteomic profile showing the expression of different sets of proteins involved in regenerative processes and thus different therapeutic properties. This also highlights their coordinated activity on tissue homeostasis and regeneration mechanisms.

## INTRODUCTION

Nowadays, it is widely accepted that the therapeutic effects of mesenchymal stromal cells (MSCs) are, at least in part, mediated by a secretome composed of paracrine soluble and insoluble functional factors, which emerge as promising candidates in regenerative medicine.

The source of MSCs can vary and includes bone marrow, adipose tissue, and extra-fetal/perinatal tissues (e.g., Wharton’s jelly, amniotic membrane, cord blood), with possible variation in the composition of the secretome.

Several studies regarding the proteomics of whole secretome have been published. For example, Kehl *et al.*^[1]^ suggested a more angiogenic network in umbilical cord Wharton’s jelly secretome compared to adipose tissue and bone marrow secretome, due to higher concentrations of angiogenesis-related proteins. Al-Sharabi *et al.*^[2]^ identified 2,157 proteins derived from bone marrow MSC secretome involved in different biological processes (cellular organization, protein metabolism) and molecular functions (cellular/protein-binding), several key growth factors, cytokines, extracellular matrix (ECM) proteins involved in wound healing and bone regeneration. In human amniotic secretome, protein and peptide components enhancing the understanding of molecular mechanisms underlying its potent immunomodulatory and anti-inflammatory actions were identified^[3]^. The amniotic-derived MSCs (AMCs) are well known in human and veterinary medicine for their antiapoptotic, immunomodulatory and anti-inflammatory properties^[4,5]^. However, the effect of these cells could also be attributed to their secretome, considering not only the proteomic data previously described, but also the *in vivo* results obtained by treatment of equine spontaneous tendon lesions^[4]^ with the secretome collected during *in vitro* culturing of AMCs, designated as conditioned medium (CM).

In addition to soluble factors, the secretome also contains extracellular vesicles (EVs), naturally released from cells and characterized by a lipid bilayer, without the ability to replicate^[6]^. The EVs are typically classified into three subtypes according to sizes and biogenesis mechanisms: exosomes (50-150 nm), microvesicles or shedding vesicles (100-1,000 nm), and apoptotic bodies (500-5,000 nm). MSCs secrete EVs with peculiar properties such as biocompatibility, low immunogenicity and ability to cross biological barriers^[7-9]^. They target specific cells and thus they can also be used to finely deliver drugs^[10-12]^.

In human medicine, many pre-clinical studies have reported the possible therapeutic potential of EVs in various diseases, including cancer, cardiovascular diseases and neurodegenerative disorders^[13,14]^. In veterinary medicine, EVs have been used for their ability to restore proper communication for a successful embryo implantation^[15]^ and to resolve breeding-induced endometrial inflammation^[16]^ *in vivo* in non-experimental animals.

Within their lipidic bilayer, EVs encapsulate a diverse array of bioactive molecules, including proteins, lipids, and nucleic acids^[17]^ which may modulate the biological functions of recipient cells. However, as the underlying cellular and molecular mechanisms remain incompletely characterized, most studies have predominantly focused on EV-associated RNA cargo - such as microRNA (miRNA), long noncoding RNA (lncRNA), and circular RNA (circRNA)^[18,19]^ - which may not fully represent the protein-mediated mechanisms that govern the majority of intercellular communication processes^[20-22]^. Increasing evidence suggests that the functional effects of MSC-derived EVs are likely attributable, at least in part, to their protein cargo rather than to RNA content alone.

It is assumed that the efficacy of a therapy depends largely on the proteomic composition of the product used. Considering the different sources of MSCs, the composition of CM in its entirety (soluble factors plus EVs) can differ from that of EVs alone. Comparing the proteomic profiles of MSCs, with CM or EVs may provide a valuable basis for selecting the most appropriate product to meet specific tissue healing or regeneration needs. In this context, some studies explored the proteomic content comparing CM and EVs from adipose MSCs and dermal fibroblasts that represent promising tools for therapeutic applications^[23]^. The proteomic analysis recognized CM and EVs as separate groups, with CM enriched in proteins of endoplasmic reticulum, Golgi apparatus and lysosomes, and EVs made of large amounts of GTPases, ribosomes and translation factors.

Many other papers were also focused on specific proteomic content of only EV-derived MSCs (MSC-EV) (see review^[24]^) for their promising diagnostic and therapeutic potential. The MSC-EV proteins contain both markers of MSCs and EVs, as well as signaling molecules regulating the self-renewal and differentiation capacities of MSCs. In addition, the list included proteins participating in cell proliferation, adhesion, migration, and morphogenesis^[25]^.

The need to understand the mechanisms of action of MSCs has led to numerous proteomic studies of these cells and the comparison with their EVs, showing that the MSC-EV proteome reflects features of both EVs and MSCs. Eirin *et al.*^[26]^ reported the finding of 5,469 proteins in adipose-derived pig MSCs and 4,937 in MSC-EVs. In differential expression analysis, EVs are richer in proteins associated with MSC-mediated tissue regeneration such as angiogenesis coagulation, apoptosis, inflammation, and ECM remodeling compared to MSCs but devoid of nuclear proteins.

Among the MSCs, extra-fetal-derived MSCs appear as appealing candidates for future clinical applications because of the safety and ethical concerns associated with pluripotency stem cells, compared to the disadvantages of adult stem cells, such as their limited natural number, harvesting challenges, and restricted expansion potential^[27]^. In this last decade, equine AMCs (eAMCs) and their EVs are gaining importance for their potential applicative use in reproductive disease^[15,16]^, but to date there is no information about their protein content. In this context, this report aimed to deepen the proteomic characterization of both AMCs and their EVs to better elucidate the molecular determinants driving their therapeutic potential. By clarifying the protein signatures associated with these regenerative products, this study may offer insights to guide a rational selection of AMC- and EV-based therapies for diverse clinical indications.

## METHODS

### Reagents

All reagents were purchased from Sigma-Aldrich (Milan, Italy), while test tubes and culture plates were purchased from Euroclone (Milan, Italy).

### Experimental design

The study comprised the isolation and culture of eAMCs, assessment of their morphology and differentiation capacity, and analysis of mesenchymal markers. All analyses were performed on pooled AMCs derived from healthy mares. From cell culture, CM was collected and EVs were isolated. Finally, proteomic analyses were performed on both pooled AMCs and pooled EVs.

### Amniotic mesenchymal stromal cell isolation and culture

Three allanto-amniotic membranes were collected after spontaneous and physiological deliveries following the protocol of Lange-Consiglio *et al.*^[28]^.

Each amnion membrane was detached from the underlying allantois membrane and fragmented into small sections. Amniotic fragments were firstly kept in phosphate-buffered saline (PBS) containing 2.4 U/mL dispase (Becton Dickinson & Company, Milan, Italy) for 9 min at 38.5 °C and then incubated in high-glucose Dulbecco’s modified Eagle’s medium (HG-DMEM) (supplemented with 10% fetal bovine serum (FBS) and 2 mM L-glutamine, Euroclone) for 5-10 min at room temperature and digested with 1 mg/mL collagenase type I and 20 μg/mL DNase (Roche, Mannheim, Germany) for 3 h at 38.5 °C. The result of the enzymatic digestion was filtered with a 100 μm filter and eAMCs were collected by centrifugation at 250 × *g* for 10 min.

AMCs were plated at a density of 1 × 10^5^ cells/cm^2^ for the first passage and at 1 × 10^4^ cells/cm^2^ for subsequent passages. Cells were cultured until passage 3 (P3), in a complete HG-DMEM medium supplemented with 10% FBS, 0.25 μg/mL amphotericin B, penicillin [100 international units per milliliter (IU/mL)], streptomycin (100 μg/mL), 2 mM L-glutamine and 10 ng/mL epidermal growth factor and incubated at 38.5 °C with 5% CO_2_ and 90% humidity.

### Amniotic mesenchymal stromal cell characterization

#### Cytology and immunocytochemistry

For the cytological analysis, the cells were seeded on glass coverslips placed into a six-well plate at a density of 1 × 10^4^ cells/cm^2^ for each well at P3. After approximately 3 days of culture and after having verified at the microscope that cell proliferation had occurred (by identification of adherence to the slide), these cells were morphologically characterized through May-Grünwald Giemsa staining.

The phenotype of the cells was then verified by immunocytochemistry. Following acetone fixation at -20 °C for 15 min, immunocytochemistry was performed automatically using the Thermo Scientific Autostainer 480S System (Thermo Fisher Scientific, Fremont, CA, USA). Endogenous peroxidase was blocked with 3% H_2_O_2_ for 10 min at room temperature. Nonspecific protein binding was prevented with 10% normal horse serum for 30 min at room temperature. Slides were incubated for 1 h at room temperature with anti-vimentin (mouse monoclonal, clone 3B4, Dako, Glostrup, Denmark) and anti-pancytokeratins (mouse monoclonal, clones AE1/AE3, Dako, Glostrup, Denmark) primary antibodies diluted 1:1,000. The slides were incubated with a biotinylated horse anti-mouse immunoglobulin G (IgG) (Vector Laboratories, Burlingame, CA, USA) diluted 1:200. Labeling was performed with Vectastain Elite ABC-Peroxidase kit (Vector Laboratories, Burlingame, CA, USA) diluted 1:150, and the reaction was visualized with Peroxidase ImmPACT DAB (diaminobenzidin) Substrate (Vector Laboratories, Burlingame, CA, USA). Slides were then counterstained with Mayer’s hematoxylin and mounted with Micromount (Diapath, Martinengo, Italy).

#### Differentiation assay

Cells at P3 were seeded at a density of 3 × 10^3^/cm^2^ for all differentiation studies.

Osteogenic differentiation was assessed by incubating cells for up to 3 weeks at 38.5 °C under 5% CO_2_ in modified Romanov *et al.*^[29]^ medium. Non-induced control cells were cultured for the same time in standard control medium. Osteogenesis was assessed by conventional von Kossa staining, using 1% silver nitrate and 5% sodium thiosulphate, which allowed detection of calcium deposits.

For adipogenic differentiation, near-confluent cells were cultured through three cycles of induction/maintenance to stimulate adipogenic differentiation. Each cycle consisted of feeding AMCs with supplemented adipogenesis induction medium, followed by culture for 3 days (38.5 °C, 5% CO_2_) and subsequent culture for another 3 days in supplemented adipogenic maintenance medium^[29]^. Non-induced control cells were cultured for the same time in standard control medium. Adipogenesis was assessed using conventional oil red O staining (0.1% in 60% isopropanol) to visualize lipid droplets.

Chondrogenic differentiation was assessed in monolayer culture by incubating cells for 3 weeks in Soncini *et al.*^[30]^ medium. Non-induced control cells were cultured for the same time in standard control medium. The presence of metachromatic matrix was demonstrated by Alcian blue staining, pH 2.5.

#### RNA extraction and RT-PCR analysis

The expression of specific markers (CD44, CD166, CD105), the hematopoietic marker CD34, and the immunogenic antigen major histocompatibility complex II (MHC-II) was investigated by reverse transcriptase polymerase chain reaction (RT-PCR) in undifferentiated cells. Total RNA was extracted at P3 from AMCs, using TrizolW reagent (Invitrogen), followed by DNase treatment according to the manufacturer’s specifications. RNA concentration and purity were measured using a NanoDrop spectrophotometer (NanoDropW ND1000, NanoDrop Technologies, Wilmington, DE, USA). Complementary DNA (cDNA) was synthesized from 200 ng total RNA, using the iScript retrotranscription kit (Bio-Rad Laboratories, Hercules, CA, USA). Conventional PCR was performed in a 25 µL final volume with DreamTaq DNA Polymerase (Fermentas, St. Leon Rot, Germany). Equine-specific oligonucleotide primers were designed using open source PerlPrimer software v. 1.1.17, based on available National Center for Biotechnology Information (NCBI) *Equus caballus* sequences or on mammal multi-aligned sequences. Primers were designed across an exon-exon junction to avoid DNA amplification. Primers were used at a final concentration of 200 nM, and their sequences are listed in Table 1. GAPDH (glyceraldehyde-3-phosphate dehydrogenase) was employed as a reference gene.

**Table 1:** Oligonucleotide sequences used for RT-PCR analysis

**Markers**	**Sequences (5’3’)**	**Product size** **(bp)**	**Annealing temperature (**°C**)**
Glyceraldehyde-3-phosphate dehydrogenase (*GAPDH*)	S: AGATCAAGAAGGTGGTGAAG A: TTGTCATACCAGGAAATGAGC	168	60
CD34 molecule (*CD34*)	S: CAGAAATTCCCAGCAAGCTC A: ATAGCAAATGAGGCCCAAGA	207	56
Integrin β-1 (*CD29*)	S: CTTATTGGCCTTGCATTGCT A: TTCCCTCGTACTTCGGATTG	184	63
CD44 antigen (*CD44*)	S: ATCCTCACGTCCAACACCTC A: CTCGCCTTTCTTGGTGTAGC	165	63
ALCAM (*CD166*)	S: CCGTTCACTATTTGGATTTGT A: CGTTTCACAGACATAGTTTCC	199	55
Endoglin (*CD105*)	S: AAGAGCTCATCTCGAGTCTG A: TGACGACCACCTCATTACTG	162	56
Major histocompatibility complex II (*MHC-II*)	S: TCTACACCTGCCAAGTG A: CCACCATGCCCTTTCTG	178	55

RT-PCR: Reverse transcriptase polymerase chain reaction; ALCAM: activated leukocyte cell adhesion molecule.

### Conditioned medium production

At P3, cell medium was changed to serum-free culture medium maintaining the same atmospheric conditions. CM was obtained by collecting the cell medium every 24 h and replacing it with fresh serum-free medium, for 3 days. To remove possible detached cells, CM was centrifuged at 1,600 × *g* for 20 min and at 4,500 × *g* for 20 min to remove debris. Finally, CM was stored at -80 °C until EV isolation.

### Extracellular vesicle isolation and characterization

EVs were isolated by ultracentrifugation of CM at 100,000 × *g* (Beckman Coulter OptimaX, Milan, Italy), 4 °C for 1 h and the obtained pellet was resuspended in serum-free medium. Vesicles were characterized according to Minimal Information for Studies of Extracellular Vesicles (MISEV) guidelines^[31]^.

#### Nanoparticle tracking analysis

EV size and concentration were assessed by nanoparticle tracking analysis (NTA) performed using a NanoSight NS300 system (Malvern Technologies, Malvern, UK), configured with a 532 nm laser, following the manufacturer’s instructions. A syringe pump with constant flow injection was used, and three videos of 60 s each were captured and analyzed with NTA software update v3.44. From each video, the mean, mode and median EV sizes were used to calculate sample concentration expressed in nanoparticles/mL.

#### Western blot and BCA assay

This analysis was performed to identify specific markers typical of EVs. A volume of 32 μL of each sample was treated with 8 μL of Laemmli buffer under reducing conditions, and the mixture was heated at 95 °C for 10 min. The sample was loaded onto a sodium dodecyl sulfate-polyacrylamide gel electrophoresis (SDS-PAGE, 4%-20%, Mini-PROTEAN TGX Precast Protein Gel, Bio-Rad) and run under an electric field before being transferred to a nitrocellulose membrane (Bio-Rad, Trans-Blot Turbo, Milan, Italy). A blocking step was performed to saturate nonspecific sites: 1 h with 5% (w/v) bovine serum albumin (BSA) in T-TBS (tris-buffered saline: 150 mM NaCl, 20 mM Tris-HCl, pH 7.4 and 0.5% Tween 20). After, the membranes were incubated overnight at 4 °C on an orbital shaker with primary antibodies, polyclonal antibody anti-tumor susceptibility gene 101 (TSG101) (1:500 dilution, Invitrogen, Monza, Italy), monoclonal antibody anti-Alix (1:1,000 dilution, Santa Cruz, CA, USA), monoclonal antibody anti-CD81 (1:500 dilution, Santa Cruz, CA, USA) and monoclonal antibody anti-CD63 (1:500 dilution, BD Biosciences New Jersey, USA). Strips were washed for 5 min, 3 times with Tris-buffered saline with Tween 20 (TBS-T), after which membranes were incubated with horseradish peroxidase (HRP)-conjugated, anti-mouse secondary antibody (Bio-Rad) diluted 1:3,000 in TBS-T with 1% BSA. Final washes were performed, and the signal was detected using Bio-Rad Clarity Western ECL Substrate (Bio-Rad) and imaged using a Chemidoc XRS+ (Bio-Rad).

To ensure the purity of the EV sample, comparative quantification of total protein [via bicinchoninic acid (BCA) assay] and Western blot analysis between EVs and AMCs was performed to assess the absence of Calnexin, typical marker for cell lysates. A pellet sample of P3 AMCs (10^6^ cells) was lysed using radio-immunoprecipitation assay (RIPA) buffer (Thermo Fisher) supplemented with Halt Protease Inhibitor (1×) according to the manufacturer’s protocol. A sample of EV suspension was mixed with RIPA buffer at a 1:1 ratio and supplemented with Halt Protease Inhibitor (1×). Samples were briefly sonicated and gently mixed for 30 min, then centrifuged at 14,000 × *g* for 15 min. Supernatants were transferred to fresh vials and protein concentration was determined using the BCA assay (Pierce^TM^). Supernatants were diluted 1:25 for the AMC sample and 1:5 for the EV sample prior to measurement. After correcting for dilution, the total protein concentration was 2,146.6 µg/mL for the AMC sample and 458.2 µg/mL for the EV sample. Both samples were adjusted to the same concentration using Laemmli buffer (5×, reducing conditions) to allow loading of 16 µg of total protein per lane. A volume of 10 µL of Precision Plus All-Blue protein standards (Bio-Rad) and 45 µL of each prepared sample were loaded per lane onto Bio-Rad TGX gradient gels (4%-15%), separated by SDS-PAGE at 220 V, and Western blotting was performed as previously described. Primary antibodies were used to detect the Calnexin marker (Sigma; 1:1,000) as a purity control for the EV sample. After three washes (5 min each) in TBS-T, membrane was incubated for 1 h with HRP-conjugated secondary antibody (Goat anti-mouse, Jackson ImmunoResearch; 1:2,500 in 1% BSA/TBST) under agitation, followed by three additional TBST washes. Chemiluminescent signals were developed using ECL substrate (Bio-Rad) and imaged on a ChemiDoc^TM^ SRX system (Bio-Rad).

#### Transmission electron microscopy

This analysis was performed by applying a 10 µL drop of EVs (20 × 10^9^ particles/mL) onto 300-mesh formvar/carbon copper grids. EVs were then fixed with a solution containing 2.5% glutaraldehyde for 5 min. After repeated washings in distilled water, the grids were contrasted with 2% uranyl acetate, air-dried, and examined using a transmission electron microscope. Digital images were acquired.

### Proteomic analysis of amniotic mesenchymal stromal cells and derived extracellular vesicles

#### High-resolution mass spectrometry analysis (nLC-HRMS)

At P3, AMCs were detached by trypsinization and centrifuged at 250 × *g* for 10 min. The resulting pellet was washed twice in PBS and centrifuged again to obtain a dry cell pellet that was stored at -80°C for subsequent protein extraction.

All samples were analyzed at UNITECH OMICs (University of Milan, Italy) using a Dionex Ultimate 3000 nano-liquid chromatography (nano-LC) system (Sunnyvale, CA, USA) connected to an Orbitrap Exploris^TM^ 240 mass spectrometer (Thermo Scientific, Bremen, Germany) equipped with a nano-electrospray ion source. Peptide mixtures were pre-concentrated on an Acclaim PepMap 100 C18 (100 μm × 2 cm, Thermo Scientific) and separated on an EASY-Spray column ES900 (25 cm × 75 μm ID) packed with Acclaim PepMap RSLC C18 (3 μm, 100 Å, Thermo Scientific) using mobile phase A (0.1% formic acid in water) and mobile phase B (0.1% formic acid in acetonitrile, 20:80 v/v) at a flow rate of 0.300 μL/min. The temperature was set to 35 °C, and the sample was injected in triplicate. Two blanks were run between samples to prevent carryover. Mass spectra were collected over an m/z range of 375-1,500 Da at 120,000 resolution, operating in data-dependent mode with a 3 s cycle time between master scans. Higher-energy collisional dissociation (HCD) was performed with a collision energy of 35 eV. Polarity: positive.

#### Bioinformatic analysis

The raw data files were analyzed using MSFragger-DDA+ (data-dependent acquisition mode, version 4.1)^[32]^ coupled with FragPipe (version 22.0) and uniprotkb equus_caballus_reviewed database (20.02.2025) to obtain protein identifications at a false discovery rate (FDR) of 0.01 and their respective label-free quantification values using the recommended parameters. Statistical analysis was performed based on the combined_protein.tsv file using Fragpipe Analyst^[33]^. Contaminant proteins were filtered out and, for the quantitation, proteins with < 2 peptides (and < 90% non-missing values) have been removed. Differential expression analysis was performed using the Limma R package, which employs feature-wise linear models combined with empirical Bayesian statistics (moderated t-test). *P*-values were adjusted for multiple testing using the Benjamini-Hochberg method. A cutoff of the adjusted *P*-value of 0.05 along with a |log2 fold change| of 1 has been applied to determine differentially expressed proteins in the comparison. Functional protein analysis and visualization has been performed in RStudio 2025.05.0 Build 496 (Posit Software, PBC, Boston, MA, USA) using a custom r script and libraries “readr”, “dplyr”, “tidyr”, “ggplot2”, “pheatmap”, “RColorBrewer”, “ggrepel”, “patchwork”, clusterProfiler, org.Hs.eg.db, ReactomePA, Stringr, gridExtra and enrichplot.

### Cross-species proteomic comparison

To evaluate the conservation of EV protein cargo across species, the complete proteomic dataset from eAMC-derived EVs (*n* = 768 proteins) was compared with publicly available EV proteomes from bovine and human mesenchymal stem cells (MSCs) retrieved from the ExoCarta database (version 6; http://www.exocarta.org/)^[34]^. The equine EV dataset included both proteins shared with eAMCs (*n* = 697) and proteins exclusive to EVs (*n* = 71), representing the complete EV proteome identified with > 2 peptides.

Proteins were matched across species based on gene symbol annotation. Venn diagrams were generated using matplotlib-venn (v0.11.9) in Python 3.11 to visualize unique and shared proteins. The 259 proteins present in all three species (conserved proteins) were functionally categorized based on Gene Ontology (GO) and literature-based classification into: Tetraspanins, endosomal sorting complexes required for transport (ESCRT)/multivesicular body (MVB) machinery, cytoskeletal proteins, membrane proteins, annexins, heat shock proteins, ECM proteins, and others. A heatmap visualized 24 representative conserved EV markers.

## RESULTS

### Amniotic mesenchymal stromal cell characterization

#### Cytology and immunocytochemistry

**Figure 1:**
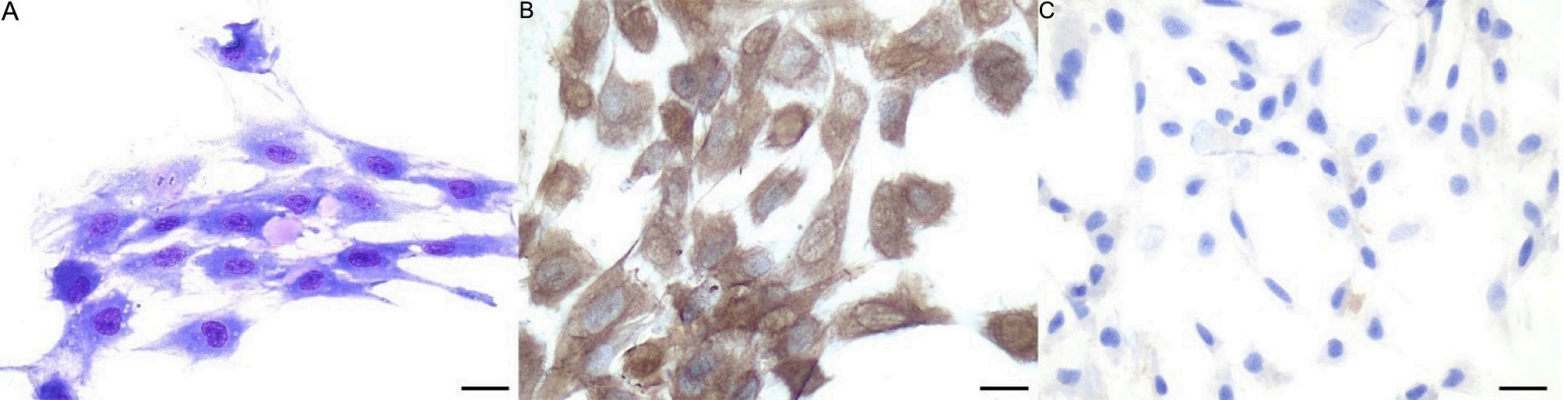
Morphology of eAMCs. (A) May-Grünwald Giemsa staining. Note the spindle morphology and the presence of extracellular matrix; (B) eAMCs are positive to vimentin and (C) negative to pancytokeratins, confirming their mesenchymal phenotype. Magnification 40×; scale bar = 25 μm. eAMCs: Equine amniotic mesenchymal cells.

At P3, eAMCs assume spindle, fibroblast-like morphology, with occasional presence of extracellular pinkish material consistent with ECM [Figure 1A]. Immunocytochemical characterization demonstrated the mesenchymal phenotype of the cells [Figure 1B] and their negativity to epithelial markers [Figure 1C].

#### Differentiation assay

**Figure 2:**
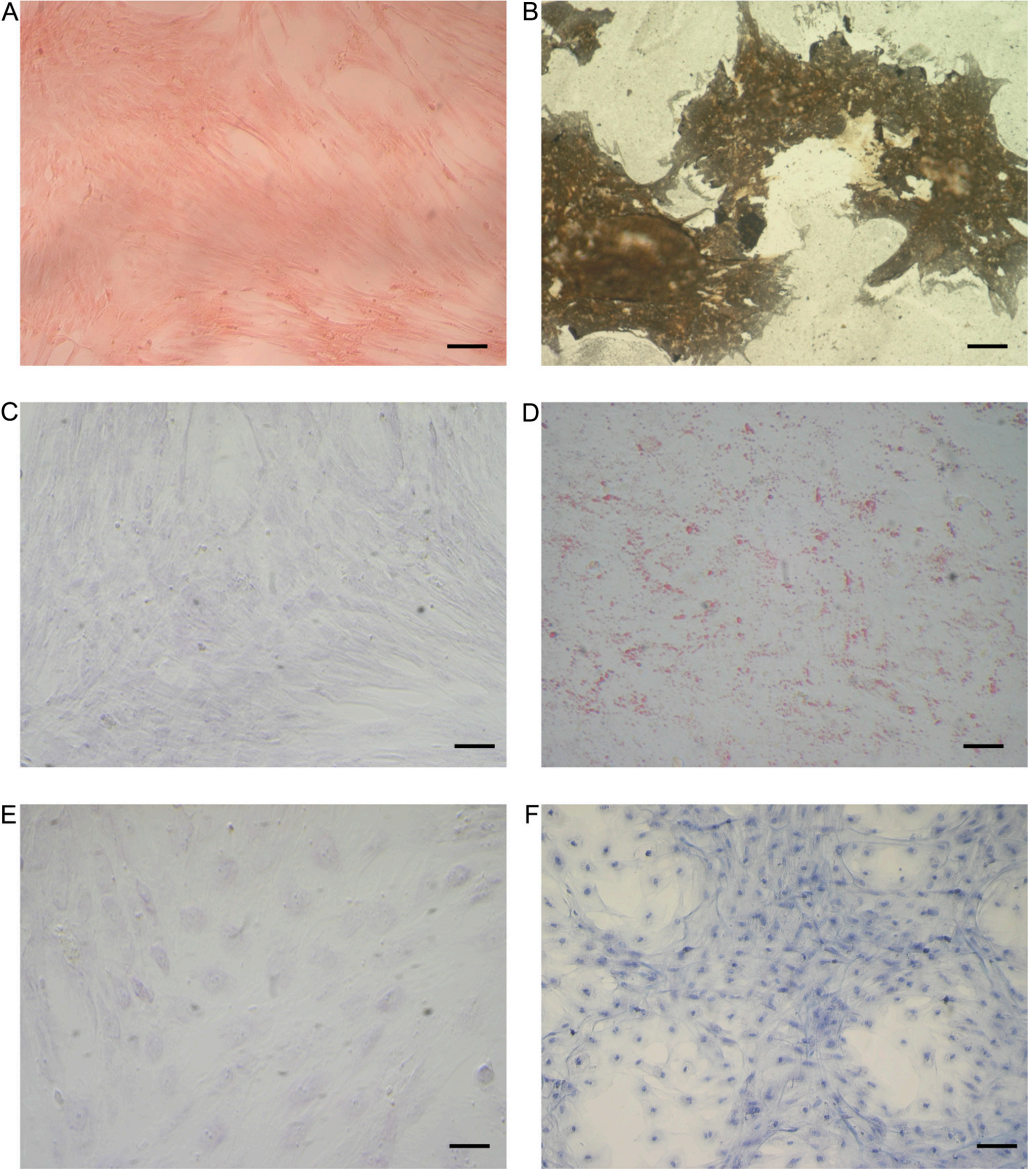
Staining of differentiated and control undifferentiated eAMCs. (A and B) von Kossa staining after osteogenic induction; (C and D) Oil red O cytoplasmic neutral lipids after adipogenic induction; (E and F) Alcian blue staining after chondrogenic induction. Magnification 20×; scale bar = 20 μm. eAMCs: Equine amniotic mesenchymal cells.

After 21 days of induction, osteogenic differentiation was confirmed by von Kossa staining, which was negative in the control cells [Figure 2A and B].

Amnion-derived cells were able to undergo adipogenic differentiation, as demonstrated by the development of positive staining for Oil Red O after 3 weeks of culture in adipogenic induction medium, while cells maintained in regular control medium showed no lipid deposits [Figure 2C and D].

Chondrogenic differentiation of eAMCs was identified by marked deposition of glycosaminoglycans in the matrix, which was observable after Alcian blue staining [Figure 2E and F].

#### RNA extraction and RT-PCR analysis

**Figure 3:**
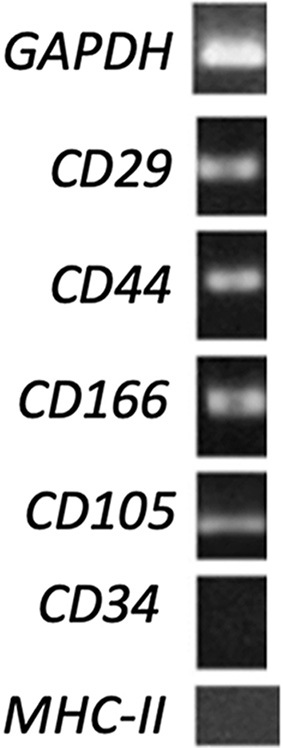
RT-PCR analysis of mesenchymal, hematopoietic and histocompatibility complex gene expression on eAMCs. GAPDH was used as reference gene. As reported in Table 1, expected product sizes: GAPDH 168 bp; CD34 207 bp; CD29 184 bp; CD44 165 bp; CD166 199 bp; CD105 162 bp; MHC-II 178 bp. Annealing temperatures: GAPDH 60 °C; CD34 56 °C; CD29 63 °C; CD44 63 °C; CD166 55 °C; CD105 56 °C; MHC-II 55 °C. RT-PCR: Reverse transcriptase polymerase chain reaction; eAMCs: equine amniotic mesenchymal cells; GAPDH: glyceraldehyde-3-phosphate dehydrogenase; MHC-II: major histocompatibility complex II.

To characterize eAMCs, a RT-PCR was set up. As previously studied^[28]^, cells expressed MSCs-specific markers (CD105, CD29 and CD44) but not hematopoietic marker (CD34), and MHC-II histocompatibility complex marker [Figure 3].

### Extracellular vesicle isolation and characterization

**Figure 4:**
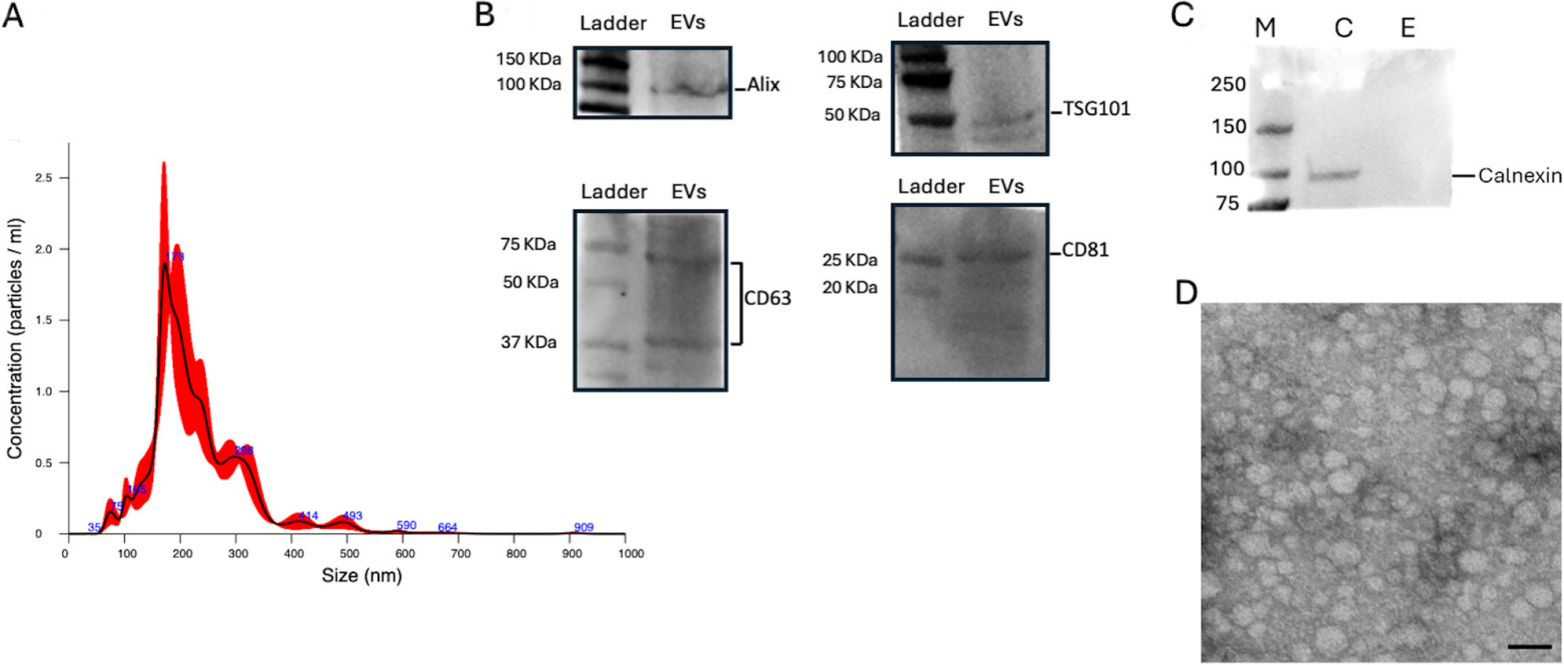
EV characterization. (A) NanoSight analysis for AMC-EV dimensions and concentrations; (B) Western blot analysis for internal marker Alix and TSG101 and surface markers CD63 and CD81, specific for EVs; (C) Comparative Western blot between AMC and EV samples for calnexin detection, typical marker for cell lysates. Legend: M = marker, C = AMC sample, E = EV sample; (D) Transmission electron microscopy image of AMC-EVs. Scale bar: 200 nm. EV: Extracellular vesicle; EVs: extracellular vesicles; AMC: amniotic-derived mesenchymal stromal cells; TSG101: tumor susceptibility gene 101.

The isolated EVs complied with the MISEV guidelines^[31]^. The NanoSight analysis revealed an average size of 229.3 ± 4.3 nm and a concentration of 1.04 × 10^11^ particles/mL. Based on the observed size distribution, the pool of isolated EVs consisted predominantly of microvesicles [Figure 4A].

Western blot analysis showed the presence of internal markers Alix and TSG101 and external markers CD81 and CD63, confirming that the preparation contained EVs [Figure 4B]. Moreover, the absence of Calnexin in the EV sample demonstrated the absence of contamination from cell lysate and the successful purification of EVs [Figure 4C].

Transmission electron microscopy (TEM) confirmed the efficiency of the isolation method for EVs, as revealed by the spheroid morphology of the EVs [Figure 4D].

### Characterization of differentially expressed proteins between eAMCs and EVs

**Figure 5:**
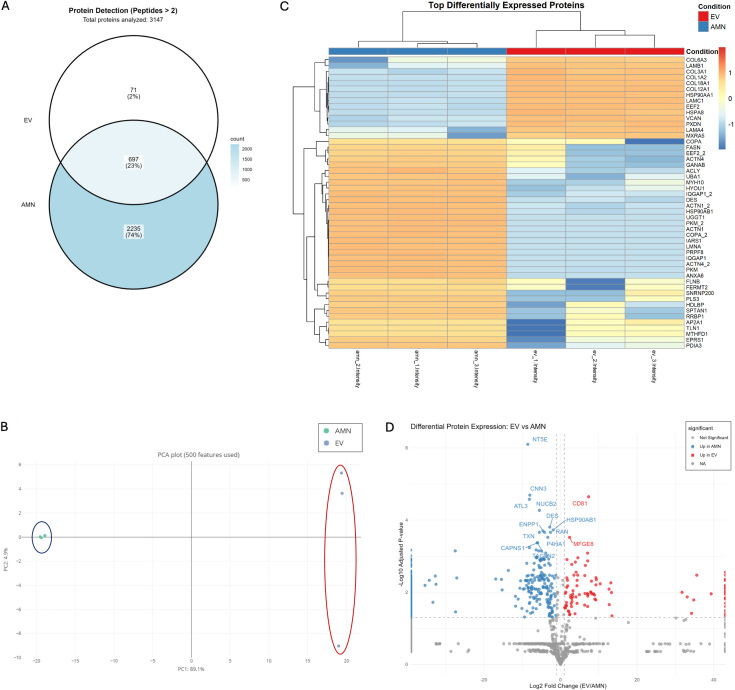
Protein expression characterization of eAMCs and EVs. (A) Venn diagram representing the proteins exclusively expressed by eAMCs (2,235 proteins, 74%), EVs (71 proteins, 2%), and those commonly detected in both eAMCs and EVs (697 proteins, 23%). Total proteins with quantifiable intensity: 3,003. Of 3,147 proteins identified with ≥ 2 peptides, 144 proteins lacking detectable intensity in both groups were excluded from the Venn diagram; (B) Principal Component Analysis (PCA) of eAMCs and EVs based on 500 protein features, demonstrating distinct clustering of the samples. PC1 (69.1% variance) and PC2 (4.0% variance) separate eAMCs (blue ellipse) from EVs (red ellipse), indicating substantial proteomic differences between parental cells and their derived vesicles; (C) Heatmap visualization of the top differentially expressed proteins between eAMCs and EVs. Clustering method: Unsupervised hierarchical clustering was performed using Euclidean distance with complete linkage on both rows (proteins) and columns (samples). Each row represents an individual protein, and each column represents a biological replicate (*n* = 3 per group). Color scale: The heatmap displays Z-score normalized protein abundances, where blue indicates lower relative expression and orange/red indicates higher relative expression. Z-scores represent the number of standard deviations from the mean abundance of each protein across all samples. The dendrogram on the left shows protein clustering relationships; (D) Volcano plot of differentially expressed proteins between EVs and eAMCs. The x-axis shows log2 fold change (EV/AMN), while the y-axis shows -log10 adjusted *P*-values. Red dots indicate proteins significantly enriched in EVs (up in EV), blue dots indicate proteins enriched in eAMCs (up in AMN), and grey dots represent proteins without significant differential expression. Significance thresholds: adjusted *P*-value < 0.05 and |log2 fold change| ≥ 1. Selected protein names are labelled for key differentially expressed proteins. eAMCs: Equine amniotic mesenchymal cells; EVs: extracellular vesicles; EV: extracellular vesicle; AMN: amniotic mesenchymal cells; NA: not applicable.

A total of 3,631 proteins were identified. Of these, 3,147 were identified with more than two peptides [Supplementary File 1]. Among these, 3,003 proteins showed quantifiable intensity in at least one sample group; 144 proteins met the peptide identification criteria but lacked detectable intensity values in both groups and were excluded from downstream analysis. Of the 3,003 quantifiable proteins, 2,235 were exclusive to eAMCs, 697 were common to both eAMCs and EVs, and 71 were exclusive to EVs [Figure 5A].

Principal component analysis (PCA) revealed distinct clustering of the two samples, with significant separation between amniotic cells and their EVs [Figure 5B]. The normalized heatmap of the differentially expressed proteins showed a quite distinct proteomic profile between eAMCs and EVs [Figure 5C]. The significant deregulated proteins between the two groups are represented also in the volcano plot [Figure 5D].

**Figure 6:**
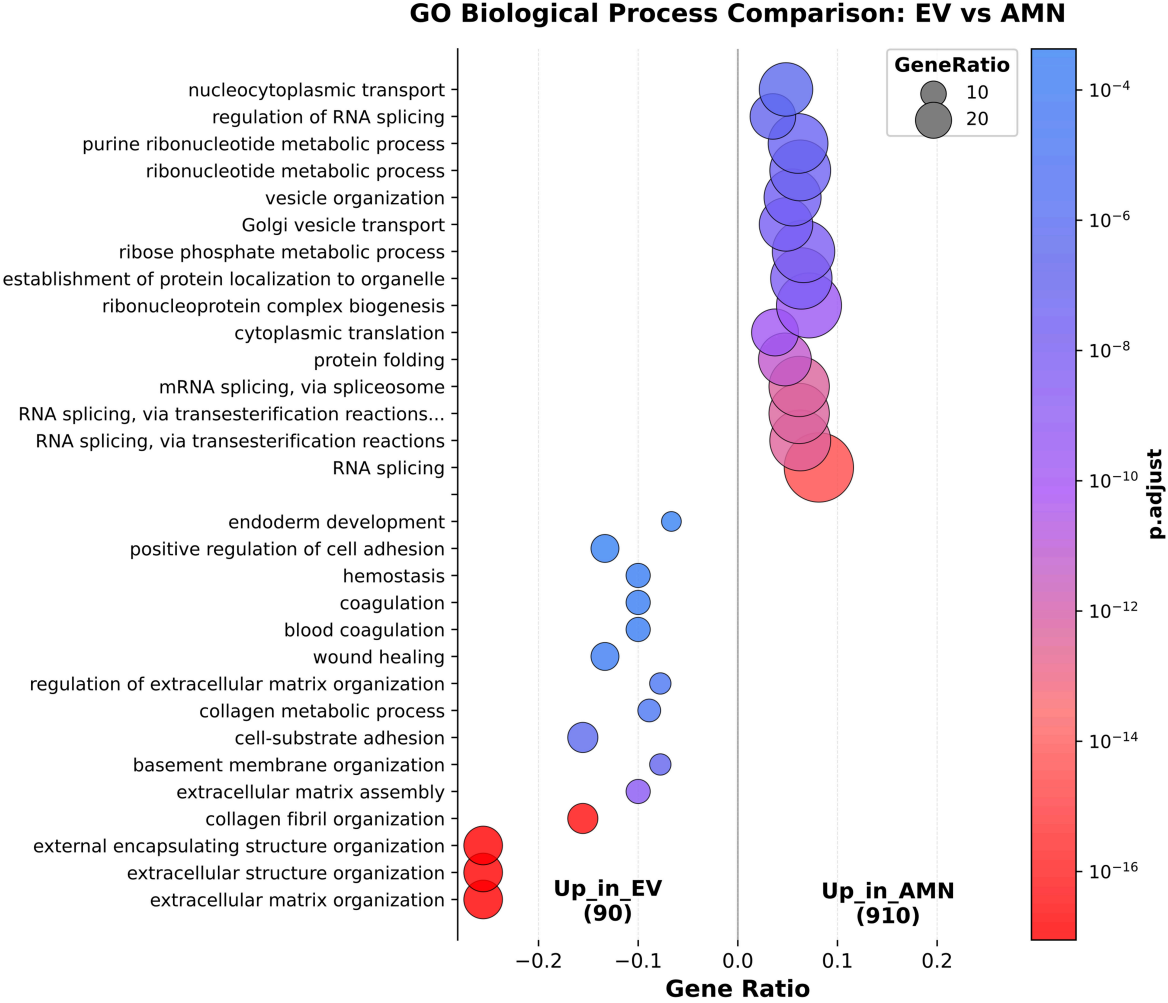
Comparison of significantly enriched Gene Ontology (GO) Biological Process terms between eAMCs and EVs. eAMCs: Equine amniotic mesenchymal cells; EVs: extracellular vesicles; EV: extracellular vesicle; AMN: amniotic mesenchymal cells.

GO enrichment analyses were performed to investigate the function of differentially expressed proteins among the two groups. eAMCs resulted enriched in biological process terms related to “RNA splicing”, “protein folding”, “Golgi vesicle transport” and others [Figure 6]. The significantly enriched terms for EVs group included “extracellular matrix organization”, “collagen fibril organization”, “extracellular matrix assembly”, “cell adhesion”, “wound healing”, “coagulation” and “homeostasis” [Figure 6]. These data are also displayed as network representation in Supplementary Figure 1.

These results suggest EVs are more enriched in “regenerative-oriented” terms compared to their parental cells, whose up-regulated proteins are mainly involved in intracellular processing pathways and metabolic activities.

The comparisons of GO Molecular Function (MF) and Cellular Component (CC)-enriched terms for eAMCs and EVs are shown in Supplementary Figures 2 and 3.

### Functional characterization of eAMC and EV differentially expressed proteins

The GO/Reactome enrichment analysis was then focused on the two groups in terms of tissue/ECM remodeling, anti-inflammatory response and regenerative pathways, to better understand the possible differences in therapeutic potential of AMCs and EVs in regenerative medicine strategies.

**Figure 7:**
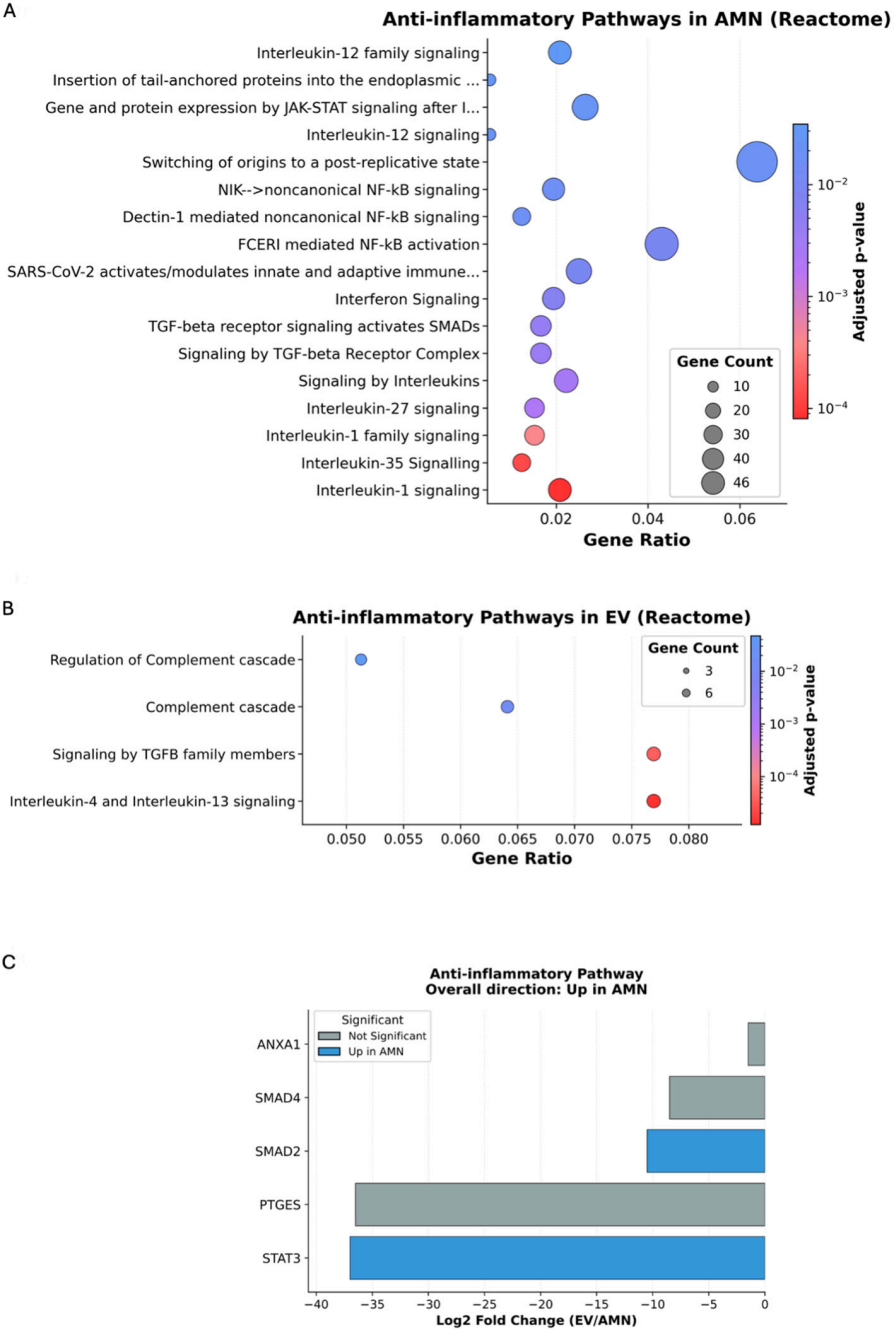
Reactome enrichment analysis for anti-inflammatory pathways. (A) Significantly enriched terms in eAMC upregulated proteins; (B) Significantly enriched terms in EV upregulated proteins; (C) Graphic representation of the overall direction of anti-inflammatory pathway regulation. eAMC: Equine amniotic mesenchymal cell; EV: extracellular vesicle; AMN: amniotic mesenchymal cells.

The results revealed that eAMCs expressed a set of proteins involved in the regulation of cytokine signaling, such as interleukin (IL)-12, IL-27, IL-1 and IL-35, nuclear factor kappa B (NF-κB), interferon (IFN) and transforming growth factor-β (TGF-β) signaling [Figure 7A]. Similar findings were obtained by GO biological process (BP), including “regulation of innate immune response” and “response to interleukin 7” [Supplementary Figure 4].

On the other hand, the terms related to anti-inflammatory pathways targeted by EV upregulated proteins included regulation of the complement cascade, signaling by TGF-β family members and IL-4 and IL-13 signaling [Figure 7B]. GO - BP-enriched terms also included “regulation of leukocyte migration”, terms related to the regulation of Janus Kinase - Signal Transducer and Activator of Transcription (JAK-STAT) signaling, and “positive regulation of inflammatory response” [Supplementary Figure 5].

However, the eAMCs group prevails in the overall direction of the anti-inflammatory pathway regulation, upregulating proteins such as Signal Transducer and Activator of Transcription 3 (STAT3) and SMAD Family Member 2 (SMAD2) [Figure 7C]. STAT3 activation results in inhibition of immune mediators and promotion of immunosuppressive factors, while SMAD2 is a downstream transcription factor of immunomodulatory TGF-β.

**Figure 8:**
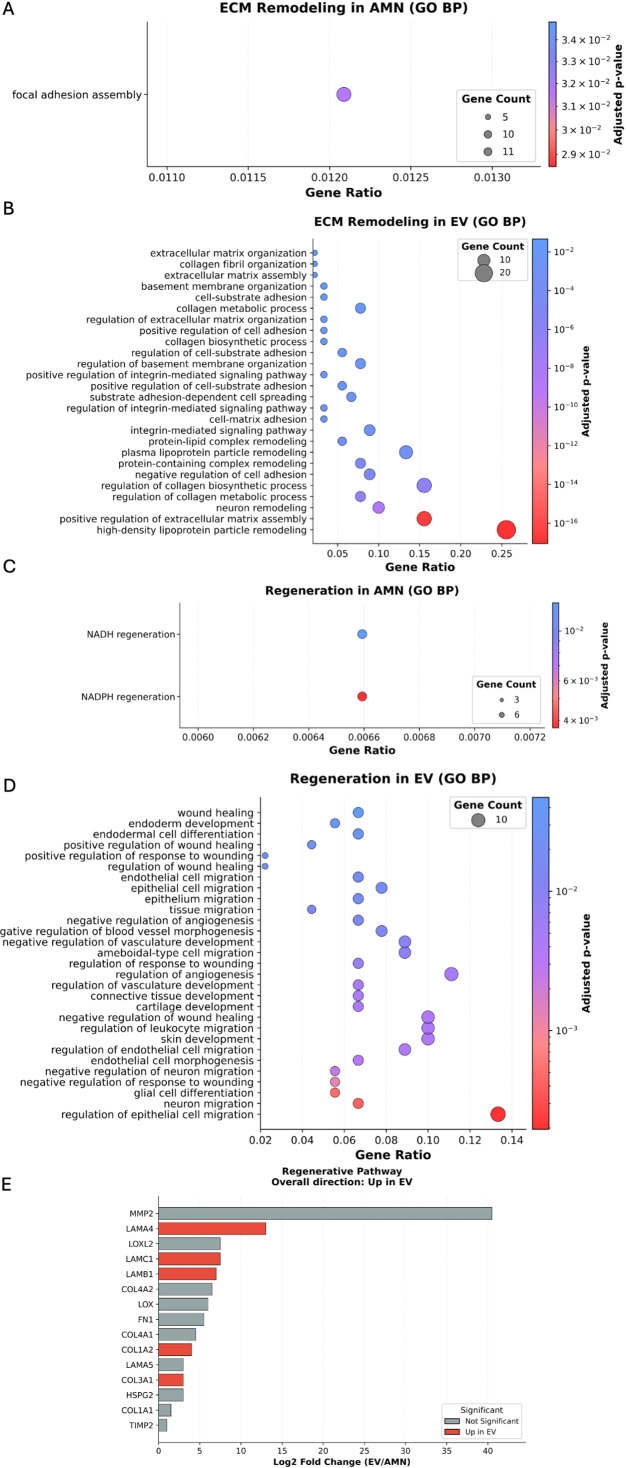
GO biological process enrichment analysis for ECM remodeling (A and B) and regenerative pathways (C-E). (A) Significantly enriched ECM remodeling terms in eAMC upregulated proteins; (B) Significantly enriched ECM remodeling terms in EV upregulated proteins; (C) Significantly enriched regeneration terms in eAMC upregulated proteins; (D) Significantly enriched regeneration terms in EV upregulated proteins; (E) Graphic representation of the overall direction of regenerative pathway regulation. GO: Gene Ontology; ECM: extracellular matrix; EV: extracellular vesicle; BP: Biological Process; eAMC: equine amniotic mesenchymal cell; AMN: amniotic mesenchymal cells.

Surprisingly, concerning ECM remodeling, the only significantly enriched term for proteins upregulated in eAMCs was “focal adhesion assembly” [Figure 8A]. On the contrary, EVs group revealed enrichment in several terms including ECM assembly and organization, collagen deposition and cell adhesion [Figure 8B]. Among the regenerative pathways targeted by eAMCs, only Nicotinamide Adenine Dinucleotide (NADH) and Nicotinamide Adenine Dinucleotide Phosphate (NADPH) regeneration terms were recognized [Figure 8C], whereas for EV proteins, terms such as “wound healing”, “angiogenesis regulation” and “cell migration” have been identified [Figure 8D].

The overall direction of regenerative pathways regulation resulted upregulated in EVs group, expressing collagen type I (COL1A2) and III (COL3A1) and laminins (LAMB1, LAMC1, LAMA4) [Figure 8E], essential proteins for tissue healing.

Finally, to better characterize the specific proteins driving the main pathways identified and to elucidate the main differences between eAMC and EV therapeutic effects, a comparison of the expression levels of proteins involved in immune regulation, oxidative resistance, ECM remodeling and angiogenesis was performed.

**Figure 9:**
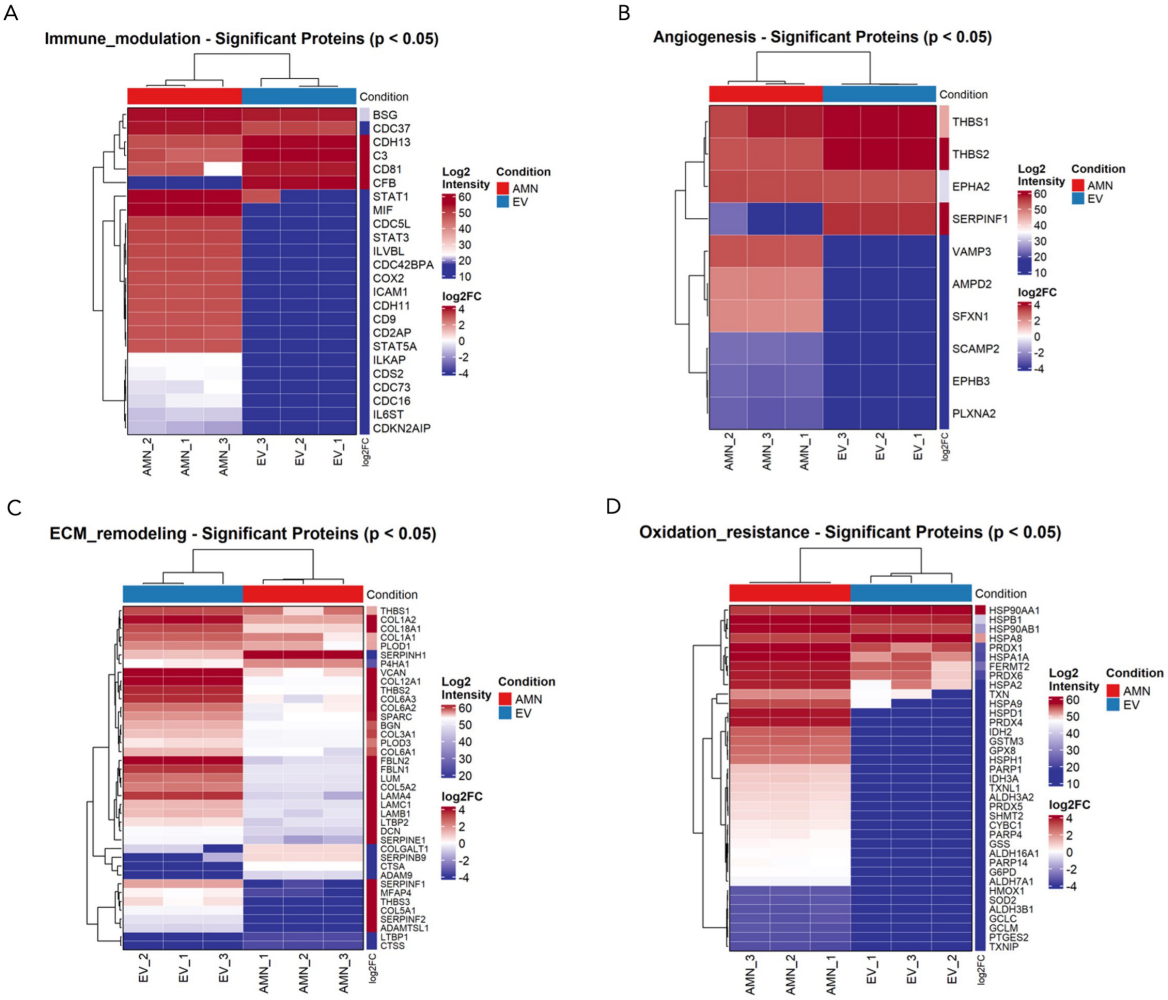
Comparison between eAMCs and EVs in terms of (A) immunomodulation, (B) angiogenesis, (C) ECM remodeling and (D) oxidation resistance. Data are presented as heatmaps representing the intensities of significantly differentially expressed proteins between eAMCs and EVs (*P* < 0.05). eAMCs: Equine amniotic mesenchymal cells; EVs: extracellular vesicles; EV: extracellular vesicle; ECM: extracellular matrix; AMN: amniotic mesenchymal cells.

Results revealed that eAMC showed higher expression of immune modulator proteins, such as transcription factor STAT3 and Cyclooxygenase-2 (COX2) enzyme, and also chemotactic adhesion-related protein ICAM1 (intercellular cell adhesion molecule-1). In contrast, EVs expressed mainly complement components C3 and complement factor B (CFB), tetraspanins such as CD81 and CDH13 cadherin, involved in recruitment, migration and adhesion of immune cells [Figure 9A]. These results suggest prevailing anti-inflammatory effects of eAMCs compared to EVs, which resulted more involved in participating in the first acute steps of immune reaction.

Among proteins relevant to angiogenesis, eAMCs upregulated proangiogenic factors such as plexin PLXNA2, sideroflexin SFXN1, ephrin receptor EPHB3 and vesicle-associated membrane protein VAMP3. Instead, EVs only expressed Serpin Family F Member 1 (SERPINF1), a protease inhibitor with anti-angiogenic activity, and thrombospondins THBS1 and THBS2, central in angiogenesis modulation [Figure 9B].

EVs also expressed a greater variety of matrix remodeling proteins compared to their parent cells. The significantly enriched proteins included the ECM-deposition protein SPARC (secreted protein acidic and rich in cysteine), fibulin glycoproteins FBLN1 and FBLN2, laminins (LAMA4, LAMB1, LAMC1), several collagens (COL1A1, COL1A2, COL3A1, COL5A1, COL5A2, COL6A2, COL6A3, COL12A1, COL18A1), and members of the serpin family, including SERPINE1, SERPINF1, and SERPINF2. In contrast, eAMCs showed significant upregulation of proteases such as cathepsins CTSA and CTSS, ADAM9 (a disintegrin and metalloproteinase domain-containing protein 9), and other serpin family members including SERPINH1 (a non-inhibitory serpin) and SERPINB9, an inhibitor of granzyme B [Figure 9C].

Concerning oxidation resistance, the only upregulated proteins for EVs were the heat-shock proteins Hsp90α (HSP90AA1, HSP90α-family class A member 1) and HSPA8. Conversely, eAMCs expressed a vast variety of antioxidant enzymes such as peroxiredoxins (PRDX 1, PRDX4, PRDX5), superoxide dismutase SOD2, glutathione peroxidase (GPX8), glucose-6-phosphate dehydrogenase (G6PD) and thioredoxins (TNX, TNXIP, TNXL1) [Figure 9D].

### Cross-species comparative analysis reveals conserved EV proteome

**Figure 10:**
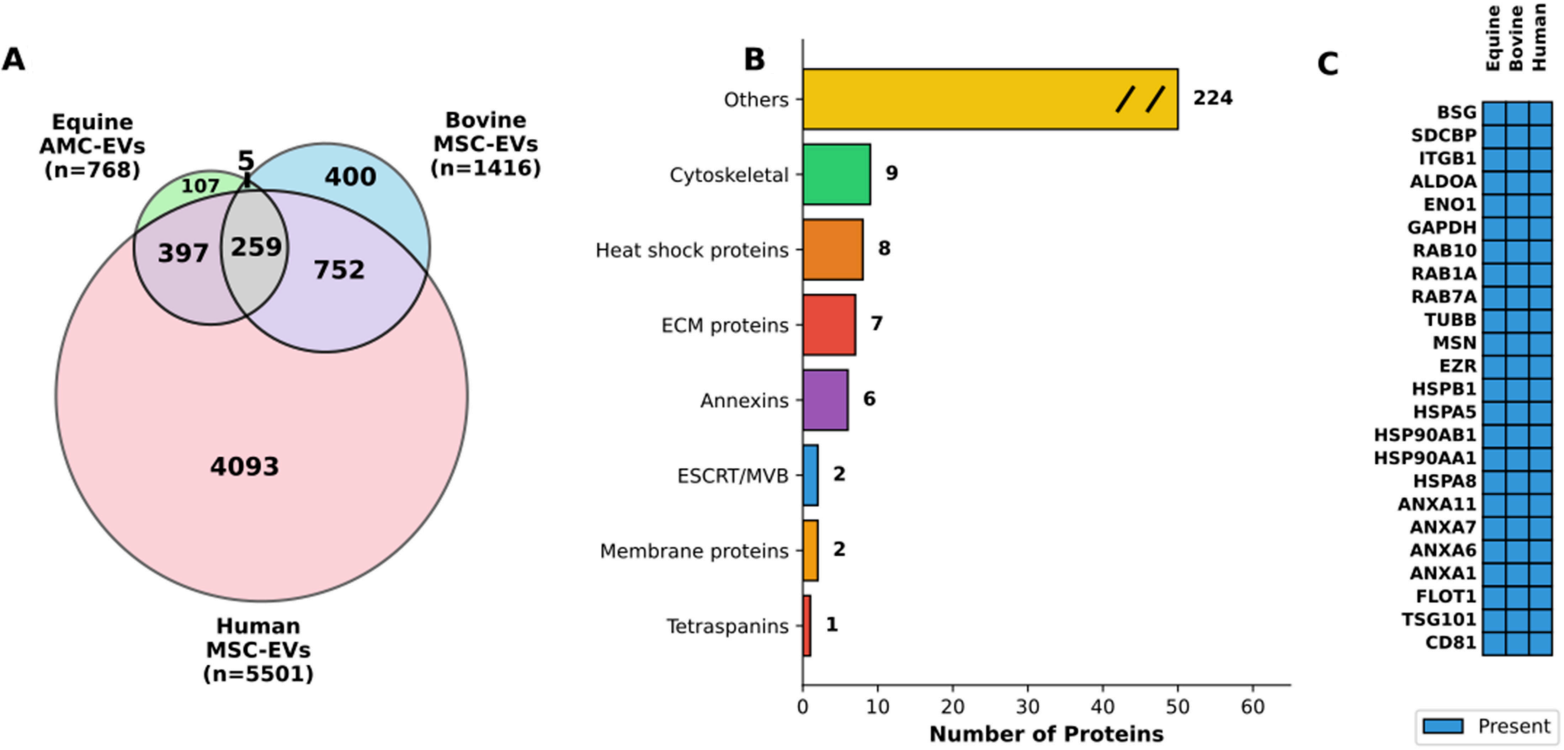
Cross-species comparative analysis of extracellular vesicle proteomes. (A) Venn diagram showing protein overlap across species. The diagram illustrates the distribution of proteins identified in EVs derived from equine AMCs (Equine AMC-EVs, *n* = 768), bovine mesenchymal stem cells (Bovine MSC-EVs, *n* = 1,416), and human mesenchymal stem cells (Human MSC-EVs, *n* = 5,501). The equine dataset comprises both proteins shared with eAMCs (*n* = 697) and proteins exclusive to EVs (*n* = 71). Numbers indicate unique and shared proteins between species, with 259 proteins conserved across all three species, representing 33.7% of the equine EV proteome; (B) Functional categorization of conserved proteins. Horizontal bar chart displaying the functional classification of the 259 proteins conserved across all three species. Categories include: Cytoskeletal proteins (*n* = 9), Heat shock proteins (*n* = 8), ECM proteins (*n* = 7), Annexins (*n* = 6), ESCRT/MVB machinery (*n* = 2), Membrane proteins (*n* = 2), Tetraspanins (*n* = 1), and Others (*n* = 224). The “Others” category is displayed with a break symbol (//) to indicate axis truncation; (C) Top conserved EV protein markers across species. Heatmap displaying 24 representative proteins conserved in EVs from all three species, organized by functional category. Blue boxes indicate presence of the protein. The panel includes: classical EV markers (CD81, TSG101, FLOT1), Annexins (ANXA1, ANXA6, ANXA7, ANXA11), heat shock proteins (HSPA8, HSPA5, HSP90AA1, HSP90AB1, HSPB1), cytoskeletal proteins (EZR, MSN, TUBB), RAB proteins (RAB7A, RAB1A, RAB10), metabolic enzymes (GAPDH, ENO1, ALDOA), and membrane proteins (ITGB1, SDCBP, BSG). EVs: Extracellular vesicles; EV: extracellular vesicle; AMCs: amniotic-derived mesenchymal stromal cells; MSC: mesenchymal stromal cell; eAMCs: equine amniotic mesenchymal cell; ECM: extracellular matrix; ESCRT: endosomal sorting complexes required for transport; MVB: multivesicular body.

To identify conserved molecular components across mammalian EV systems, comparative proteomic analysis of eAMC-derived EVs (*n* = 768 proteins) with bovine and human MSC-EV datasets from ExoCarta (*n* = 1,416 and *n* = 5,501, respectively) was performed. Venn diagram analysis revealed 259 proteins (33.7% of equine proteome) conserved across all three species, despite substantial differences in total protein numbers [Figure 10A]. Additionally, 397 proteins were shared between equine and human EVs, while 107 proteins were unique to equine EVs.

Functional categorization of the 259 conserved proteins showed enrichment in core EV-associated pathways [Figure 10B], including cytoskeletal proteins (*n* = 9), heat shock proteins (*n* = 8), ECM proteins (*n* = 7), and annexins (*n* = 6), along with classical EV markers such as tetraspanins (*n* = 1) and ESCRT/MVB machinery components (*n* = 2). The majority (*n* = 224, 86.5%) comprised diverse cellular proteins categorized as “Others”, reflecting the heterogeneous nature of EV cargo.

Detailed analysis confirmed the universal presence of canonical EV markers [CD81, TSG101, Flotillin1 (FLOT1)], calcium-binding proteins (annexins), protein quality control machinery (heat shock proteins), cytoskeletal components (EZR, Ezrin; MSN, Moesin; TUBB, Tubulin beta), vesicle trafficking regulators (RAB proteins), metabolic enzymes (GAPDH; ENO1, Enolase 1; ALDOA, Aldolase A), and membrane proteins (ITGB1, Integrin beta-1; SDCBP, Syntenin-1; BSG, Basigin) across all species [Figure 10C]. These findings demonstrate conservation of core EV molecular machinery across mammalian species, supporting the translational relevance of equine EV models.

### DISCUSSION

Many researchers have made it clear that MSCs engraft at low levels and that their therapeutic effects are not solely due to engraftment and differentiation, but may also be mediated by the release of factors that influence target cells via paracrine mechanisms^[35]^.

eAMCs and their secretome have been studied *in vivo* for tendon and reproductive diseases^[4,16]^, suggesting that cells alone, CM alone, or EVs alone can exert therapeutic effects. However, the selection of an appropriate regenerative product could contribute to the advancement of their clinical translation and application.

According to this view, this study analyzed the comparative proteomic profiles of eAMCs and their EVs to identify differences between them and to better understand their potential for developing cell-free therapeutic strategies.

First, rigorous characterization of AMCs and their EVs was essential to ensure the quality of the isolated regenerative products.

MSCs are a heterogeneous population of fibroblast-shaped cells with the *in vitro* capability to adhere to plastic, differentiate into osteoblast, adipocyte, and chondrocyte lineages, and express mesenchymal markers - but not hematopoietic or HLA-DR markers - as required by the International Society for Cellular Therapy (ISCT)^[36,37]^.

The eAMCs have previously been fully characterized^[28]^ according to the criteria of ISCT and, in this study, AMCs were successfully isolated and expanded *in vitro*, exhibiting adhesion to plastic, a spindle-shaped morphology, and expression of some well-defined MSC markers (CD29, CD44, CD166 and CD105), while they were negative for immune-histocompatibility complex II (MHC-II) and hematopoietic (CD34) markers.

By culturing AMCs under osteogenic, adipogenic and chondrogenic conditions, the differentiation potential of AMCs was investigated, confirming their differentiative potential in mesenchymal lines.

EVs were characterized following the MISEV guidelines^[31]^. Based on their dimensions detected by NTA analysis and TEM, the isolated nanoparticles can be classified as microvesicles. Moreover, they respond to internal (Alix and TSG101) and external markers (CD81 and CD63) and are negative for markers typical of cell lysates (Calnexin).

Several studies have analyzed the proteome of human AMCs and MSC secretomes^[2,38]^, but few have compared the proteomic profiles of MSCs and their secreted EVs, as previously noted by Eirin *et al.*^[26]^. Recently, Braga *et al.*^[39]^ examined the proteomic differences in bone marrow-derived MSCs and their EVs under both normoxic and hypoxic conditions; however, no direct comparison between MSCs and their EVs was performed.

In our study, 3,631 proteins were identified. Of these, 3,147 were identified with more than two peptides; 2,235 were exclusive to eAMCs, 697 were shared between eAMCs and EVs, and 71 were exclusive to EVs. The total number of proteins cannot be directly compared with other proteomic studies on MSCs and their EVs, as results are influenced by cell type, culture conditions, and experimental factors affecting secretome protein composition. Additionally, MSC cultures comprise a heterogeneous population, including differentiated cells, lineage-committed precursors, and progenitor cells, which increases complexity and variability.

However, our data underlie a distinct proteomic profile between eAMCs and EVs, with several proteins involved in tissue regeneration differentially expressed between the two samples.

From a prior GO enrichment analysis, EVs were more enriched in terms related to regenerative pathways compared to their parent cells, whose upregulated proteins are mainly involved in intracellular processing and metabolic activities. However, the higher complexity of cellular processes and the resultant abundance of proteins involved in basal homeostatic events, compared with the selective packaging of a smaller set of proteins into EVs, should be taken into account.

Given the large number of proteins/pathways predicted in our study, we focused on processes relevant to regenerative applications, including immunomodulatory and anti-inflammatory properties, oxidative stress resistance, ECM remodeling and angiogenic property.

### Immunomodulatory and inflammatory properties of eAMCs and EVs

According to pathway enrichment analysis, the more significant proteins affecting inflammatory responses expressed by eAMCs are represented by a set of molecules involved in the regulation of cytokine signaling, such as IL-12, IL-27, IL-1 and IL-35, NF-κB, IFN and TGF-β signaling. On the other hand, the terms related to anti-inflammatory pathways targeted by EV upregulated proteins included regulation of the complement cascade, signaling by TGF-β family members and IL-4 and IL-13 signaling.

IL-4 and IL-13, classically considered anti-inflammatory cytokines by virtue of their ability to inhibit type 1 inflammation (IFN-γ, IL-12, nitric oxide (NO), are secreted by T helper type 2 (Th2)-polarized T cells, granulocytes and monocytes/macrophages^[40]^. In the context of the immune system, IL-4 and IL-13 trigger Th2 T cells differentiation, M2 macrophage polarization, MHC-II expression, B cell and plasma cell differentiation and antibody isotype switch among others^[41]^.

EVs showed higher expression of complement components, including C3 and CFB. C3 is a crucial molecule in the complement system, as its cleavage represents a central step in cascade amplification, ultimately leading to the formation of the membrane attack complex (MAC), which induces cell lysis. The cleavage of C3 molecule also induces release of C3a and C5a signaling molecules, which are potent inflammation chemoattractants, promoting leukocyte recruitment, vasodilation, cytokine release and activation of adaptive^[42]^. In the alternative activation pathway, CFB is an indispensable element for the formation of C3 convertase, responsible for the cleavage of C3 molecules.

Although being known as an extracellular mechanism, the latest evidence has discovered an intracellular extrahepatic complement also in non-immune cells. This tissue-resident complement may help in mediating local inflammation^[43]^. So, it is not unbelievable to detect the presence of complement molecules also into amniotic cells and vesicles.

Although the higher expression of CD81 by EVs might not seem surprising, as this protein represents a widely used marker for EV characterization (as shown also in our results), CD81 is also well-known for its involvement in B and T cell adhesion, activation and differentiation^[44]^. This surface protein, belonging to the tetraspanins superfamily, regulates biogenesis, trafficking and membrane compartmentalization of EVs^[45]^. However, CD81 is also reported for its role in the formation of a complex with other tetraspanins, able to reduce the threshold for B cell activation and for providing a co-stimulatory signal for the activation and differentiation of T cells^[44]^.

eAMCs showed higher expression of immune modulator proteins, including STAT3 and COX2. STAT3 is an important transcription factor, regulator for T cell differentiation, up-regulating a number of genes responsible for cell survival and proliferation, anti-inflammatory IL-6, IL-10, TGF-β, vascular endothelial growth factor (VEGF) or down-regulating pro-inflammatory factors such as IFN-β, IFN-γ, IL-12, tumor necrosis factor (TNF), C-X-C Motif Chemokine Ligand 10 (CXCL10), and C-C Motif Chemokine Ligand 5 (CCL5)^[46]^. The COX2 enzyme is responsible for the production of prostaglandin E2 (PGE2), which, although generally known as a mediator of active inflammation, can also suppress acute inflammatory mediators. COX2/PGE2 contributes to inflammation resolution by suppressing the proliferation of activated lymphocytes, promoting macrophage polarization toward the M2 phenotype, and supporting the production of regulatory T cells^[47,48]^.

These data confirm our previous *in vitro* results demonstrating the ability of eAMCs to inhibit peripheral blood mononuclear cell (PBMC) proliferation after allogenic stimulation, either when co-cultured in cell-to-cell contact, or when the two cell types (PBMCs and eAMCs) are physically separated by a transwell membrane, suggesting that also soluble factors are implicated in this phenomenon^[4]^.

Additionally, the chemotactic adhesion-related protein ICAM1 was expressed by eAMCs. Typically induced during inflammatory responses, intercellular cell adhesion molecule (ICAM) significantly promotes the migration of MSCs to the inflammation site, but also emerging evidence recognizes its involvement in resolution of inflammation and wound healing^[49]^.

These results suggest complementary anti-inflammatory effects of eAMCs and their EVs, with eAMCs exhibiting chemotactic and immunosuppressive roles, while EVs show the expression of mediators involved in the first steps of acute immune response.

### Oxidative resistance of eAMCs and EVs

During injury, reactive oxygen species (ROS) are produced in high amounts, thus the balancing of redox signaling is fundamental to prevent cellular damage, metabolic alterations and chronic inflammation which finally contribute to delayed or impaired healing.

From our results, the only up-regulated proteins in EVs were HSP90AA1 and HSPA8. HSP90AA1 is involved in the regulation of the stabilization and balance between integrity and degradation of mitochondrial cytochrome p4502E1, which produces free radicals and ROS, contributing to oxidative stress^[50]^. HSPA8 belongs to the heat shock 70 kDa protein family, whose members are chaperone proteins that can modulate excessive ROS production by promoting the activity of antioxidant enzymes and the removal of free radicals^[51]^.

eAMCs expressed several antioxidant enzymes such as peroxiredoxins (PRDX 1/4/5), superoxide dismutase SOD2, glutathione peroxidase (GPX8), G6PD and thioredoxins (TNX/IP/L1). By eliminating ROS, these enzymes contribute to the balancing of redox homeostasis, essential for normal cell growth and organismal survival^[52-54]^.

According to these data, both parent cells and EVs have potential for oxidative stress modulation, but the proteins driving this mechanism differ between them, including in abundance.

### Extracellular matrix remodeling properties of eAMCs and EVs

ECM remodeling is an essential process for tissue regeneration and is regulated by the deposition of collagens, proteoglycans and the appropriate modulation of protease secretion^[55]^. In this study, the significantly enriched terms for EV group included “extracellular matrix organization”, “collagen fibril organization”, “extracellular matrix assembly”, “cell adhesion” and “wound healing”.

In this study, amniotic EVs expressed a greater variety of proteins involved in ECM deposition (SPARC) and remodeling, including glycoproteins (FBLN1 and FBLN2) and laminins (LAMA4, LAMC1, LAMB1), collagens (COL1A1, COL1A2, COL3A1, COL5A1, COL5A2, COL6A2, COL6A3, COL12A1, COL18A1) and serpins, protease inhibitors that target proteases and caspases, preventing an inadequate matrix degradation activity (SERPINE1, SERPINF1, SERPINF2).

The collagens are the most abundant components of ECM. Type I collagen is an important member of the collagen family, which is a key structural component of the ECM^[56]^.

The ECM proteins and their receptors of the integrin family have been identified as important regulators of tissue homeostasis, influencing the balance between cell renewal and differentiation^[57]^.

Laminins, a family of non-collagenous ECM glycoproteins that constitute most of the basement membrane, have been implicated in a wide variety of biological processes, including cell adhesion, differentiation, migration, and signaling.

The abundance of ECM proteins expressed by EVs corroborates the findings obtained in equine clinical applications. The presence of these proteins into EV cargo may have promoted the efficacious regeneration of spontaneous tendon lesions of sport horses treated with AMC-CM^[4]^. Although EVs are not the only component of CM, they represent an important functional part. Indeed, to further support this evidence, in the context of equine reproductive pathologies, amniotic EVs have been successfully employed to restore defective embryo-maternal communication by regenerating fibrotic endometrial tissue affected by chronic degenerative endometritis. This treatment improved endometrial competence^[19]^ and suggests that EVs contribute to *in vivo* healing alongside the soluble factors present in AMC-CM.

On the other hand, eAMCs expressed proteases including cathepsins (CTSA and CTSS), or metalloproteinase/disintegrin (ADAM9), responsible for the cleavage of several ECM components^[58]^. eAMCs also expressed other serpin family members. These include a non-inhibitory molecule that serves as a collagen chaperone (SERPINH1) and an inhibitor of granzyme B (SERPINB9), which prevents excessive apoptosis during the physiological cleavage of ECM proteins.

According to these data, EVs proteins are more matrix deposition-oriented, while eAMCs express fewer proteins which are mostly related to protease activity and active dynamic remodeling of ECM.

### Angiogenic properties of eAMCs and EVs

Among angiogenic-related proteins, EVs expressed only SERPINF1 and thrombospondins (THBS1 and THBS2). This family of ECM proteins plays important roles in modulating angiogenesis, primarily inhibiting it, although evidence also indicates pro-angiogenic activity depending on interactions with receptors and integrins^[59,60]^. SERPINF1, also known as pigment epithelial-derived factor, is a protease inhibitor central in controlling angiogenesis: thanks to its antiangiogenic properties, this serpin regulates abnormal vessel regulation that can arise from pathological stimuli and inflammatory conditions, with evidence in its tumor-regression activity^[61]^.

On the contrary, eAMCs showed higher expression of proangiogenic proteins, such as plexin PLXNA2, sideroflexin SFXN1, which is involved in heme biosynthesis^[62]^, vesicle-associated membrane protein VAMP3, which mediates endocytic trafficking in platelets, essential for transferrin and fibrinogen intake, clot retraction, and platelet spreading^[63]^, and also ephrin EPHB3, essential for vasculature remodeling and capillary formation^[64]^.

According to our data, both eAMCs and EVs express angiogenesis-related proteins; however, the parent cells seem to mainly promote the formation of new blood vessels, whereas EVs exert a more controlling effect in restraining and averting abnormal angiogenesis.

Finally, the comparative proteomic analysis demonstrates substantial conservation of EV protein cargo across mammalian species, with 259 proteins shared among equine, bovine, and human MSC-derived EVs. This conservation encompasses essential cellular machinery including cytoskeletal components, heat shock proteins, and classical EV markers such as tetraspanins and ESCRT/MVB proteins. The enrichment of these functional categories reflects fundamental requirements for vesicle biogenesis, cargo selection, and membrane architecture that appear largely independent of species or cellular origin. However, the identification of 107 equine-specific proteins suggests the existence of species-adapted mechanisms that may confer specialized functions to EVs from different sources. The high degree of molecular similarity observed across species provides a rational basis for cross-species therapeutic applications and supports the use of large animal models, particularly equine systems, in preclinical EV research with translational objectives.

Altogether, these findings link the regenerative effects of eAMCs and their EVs, observed in our previous *in vitro* and *in vivo* studies, to proteomic evidence, highlighting potential candidate proteins that collectively contribute to the therapeutic action of these regenerative products. The positive outcome of eAMC and/or their EV administration for spontaneous tendon lesions^[5]^ and endometritis^[15,16]^ confirmed their regenerative effect, despite the lack of molecular evidence underlying their therapeutic properties.

This study provides new insights into the protein content of amniotic parent cells and their secreted EVs. Comparison shows that eAMCs and their EVs express distinct sets of proteins involved in anti-inflammatory activity, oxidative stress resistance, and angiogenesis pathways. This emphasizes their important complementary roles in the fine regulation of the main processes involved in tissue regeneration, proposing AMCs and EVs as a dynamic and effective “couple” rather than separated actors with similar effects. Consequently, to date, we are still unable to definitively elect cells over EVs (or vice versa) as a better therapeutic candidate.

The limits of this paper concern the molecular and structural heterogeneity of EVs, especially considering the multitude of variable parameters deriving from cell culturing that can influence their complexity and diversity. In all our studies, the protocol of isolation and culture of eAMCs^[28]^ and the procedure for EV ultracentrifugation^[15,18]^ were set and standardized. However, the precise moment of EV collection could influence their molecular cargo. For each of our studies, a pool of eAMCs was obtained from three amniotic membranes, yet the donors differ each time, and the isolated mesenchymal cells can comprise differentiated cells, lineage-committed precursors and progenitor cells, which increase this complexity and variability. To date, we have studied miRNA cargo and proteomic profile of eAMCs, however, although these experiments were carried out with standard and controlled procedures, the molecular response of eAMCs and their EVs may not fully replicate the complexities of *in vivo* conditions, potentially limiting the translational relevance of our findings. Our previous *in vivo* results with eAMCs and EVs highlight the therapeutic effect of these products, but their exact *in vivo* mechanism requires further analysis for therapeutic and diagnostic applications.

In conclusion, to our knowledge, this study is the first to compare eAMCs and their secreted EVs from a proteomic perspective. These two regenerative products are characterized by distinct proteomic profiles, with each expressing different sets of proteins involved in processes relevant to regenerative applications, including immunomodulation, oxidative stress resistance, ECM remodeling, and angiogenesis. While aimed at uncovering the different therapeutic properties of eAMCs and EVs, these findings also highlight their coordinated activity in tissue homeostasis and regeneration.

The proteins identified in this study that are associated with regenerative properties provide a foundation for further investigation into the functional significance and specific biological roles of eAMCs and their EVs.

## DECLARATIONS

### Availability of data and materials

The dataset supporting the conclusions of this article is deposited at https://massive.ucsd.edu/ProteoSAFe/static/massive.jsp.

### Copyright

© The Author(s) 2026.

## References

[1] Kehl D, Generali M, Mallone A (2019). Proteomic analysis of human mesenchymal stromal cell secretomes: a systematic comparison of the angiogenic potential. NPJ Regen Med.

[2] Al-Sharabi N, Gruber R, Sanz M (2023). Proteomic analysis of mesenchymal stromal cells secretome in comparison to leukocyte- and platelet-rich fibrin. Int J Mol Sci.

[3] Muntiu A, Papait A, Vincenzoni F (2023). Disclosing the molecular profile of the human amniotic mesenchymal stromal cell secretome by filter-aided sample preparation proteomic characterization. Stem Cell Res Ther.

[4] Lange-Consiglio A, Rossi D, Tassan S, Perego R, Cremonesi F, Parolini O (2013). Conditioned medium from horse amniotic membrane-derived multipotent progenitor cells: immunomodulatory activity *in vitro* and first clinical application in tendon and ligament injuries *in vivo*. Stem Cells Dev.

[5] Silini AR, Magatti M, Cargnoni A, Parolini O (2017). Is immune modulation the mechanism underlying the beneficial effects of amniotic cells and their derivatives in regenerative medicine?. Cell Transplant.

[6] Théry C, Witwer KW, Aikawa E (2018). Minimal information for studies of extracellular vesicles 2018 (MISEV2018): a position statement of the International Society for Extracellular Vesicles and update of the MISEV2014 guidelines. J Extracell Vesicles.

[7] Barile L, Lionetti V, Cervio E (2014). Extracellular vesicles from human cardiac progenitor cells inhibit cardiomyocyte apoptosis and improve cardiac function after myocardial infarction. Cardiovasc Res.

[8] Abyadeh M, Alikhani M, Mirzaei M, Gupta V, Shekari F, Salekdeh GH (2024). Proteomics provides insights into the theranostic potential of extracellular vesicles. Adv Protein Chem Struct Biol.

[9] Haghighitalab A, Dominici M, Matin MM (2023). Extracellular vesicles and their cells of origin: open issues in autoimmune diseases. Front Immunol.

[10] Crivelli B, Chlapanidas T, Perteghella S, Italian Mesenchymal Stem Cell Group (GISM) (2017). Mesenchymal stem/stromal cell extracellular vesicles: From active principle to next generation drug delivery system. J Control Release.

[11] Mehryab F, Rabbani S, Shahhosseini S (2020). Exosomes as a next-generation drug delivery system: An update on drug loading approaches, characterization, and clinical application challenges. Acta Biomater.

[12] Herrmann IK, Wood MJA, Fuhrmann G (2021). Extracellular vesicles as a next-generation drug delivery platform. Nat Nanotechnol.

[13] Shekari F, Nazari A, Assar Kashani S, Hajizadeh-Saffar E, Lim R, Baharvand H (2021). Pre-clinical investigation of mesenchymal stromal cell-derived extracellular vesicles: a systematic review. Cytotherapy.

[14] Zhang C, Deng R, Zhang G (2022). Therapeutic effect of exosomes derived from stem cells in spinal cord injury: a systematic review based on animal studies. Front Neurol.

[15] Lange-Consiglio A, Funghi F, Cantile C, Idda A, Cremonesi F, Riccaboni P (2020). Case report: use of amniotic microvesicles for regenerative medicine treatment of a mare with chronic endometritis. Front Vet Sci.

[16] Lange-Consiglio A, Gaspari G, Funghi F (2023). Amniotic mesenchymal-derived extracellular vesicles and their role in the prevention of persistent post-breeding induced endometritis. Int J Mol Sci.

[17] Doyle LM, Wang MZ (2019). Overview of extracellular vesicles, their origin, composition, purpose, and methods for exosome isolation and analysis. Cells.

[18] Lange-Consiglio A, Lazzari B, Perrini C (2018). MicroRNAs of equine amniotic mesenchymal cell-derived microvesicles and their involvement in anti-inflammatory processes. Cell Transplant.

[19] Corrado C, Barreca MM, Zichittella C, Alessandro R, Conigliaro A (2021). Molecular mediators of RNA loading into extracellular vesicles. Cells.

[20] Park H, Park H, Mun D (2018). Extracellular vesicles derived from hypoxic human mesenchymal stem cells attenuate GSK3β expression via miRNA-26a in an ischemia-reperfusion injury model. Yonsei Med J.

[21] Zhu LP, Tian T, Wang JY (2018). Hypoxia-elicited mesenchymal stem cell-derived exosomes facilitates cardiac repair through miR-125b-mediated prevention of cell death in myocardial infarction. Theranostics.

[22] Cheng H, Chang S, Xu R (2020). Hypoxia-challenged MSC-derived exosomes deliver miR-210 to attenuate post-infarction cardiac apoptosis. Stem Cell Res Ther.

[23] Niada S, Giannasi C, Magagnotti C, Andolfo A, Brini AT (2021). Proteomic analysis of extracellular vesicles and conditioned medium from human adipose-derived stem/stromal cells and dermal fibroblasts. J Proteomics.

[24] Qiu G, Zheng G, Ge M (2019). Functional proteins of mesenchymal stem cell-derived extracellular vesicles. Stem Cell Res Ther.

[25] Kim HS, Choi DY, Yun SJ (2012). Proteomic analysis of microvesicles derived from human mesenchymal stem cells. J Proteome Res.

[26] Eirin A, Zhu XY, Puranik AS (2016). Comparative proteomic analysis of extracellular vesicles isolated from porcine adipose tissue-derived mesenchymal stem/stromal cells. Sci Rep.

[27] Antoniadou E, David AL (2016). Placental stem cells. Best Pract Res Clin Obstet Gynaecol.

[28] Lange-Consiglio A, Corradetti B, Bizzaro D (2012). Characterization and potential applications of progenitor-like cells isolated from horse amniotic membrane. J Tissue Eng Regen Med.

[29] Romanov YA, Svintsitskaya VA, Smirnov VN (2003). Searching for alternative sources of postnatal human mesenchymal stem cells: candidate MSC-like cells from umbilical cord. Stem Cells.

[30] Soncini M, Vertua E, Gibelli L (2007). Isolation and characterization of mesenchymal cells from human fetal membranes. J Tissue Eng Regen Med.

[31] Welsh JA, Goberdhan DCI, O’Driscoll L, MISEV Consortium (2024). Minimal information for studies of extracellular vesicles (MISEV2023): from basic to advanced approaches. J Extracell Vesicles.

[32] Yu F, Deng Y, Nesvizhskii AI (2025). MSFragger-DDA+ enhances peptide identification sensitivity with full isolation window search. Nat Commun.

[33] Hsiao Y, Zhang H, Li GX (2024). Analysis and visualization of quantitative proteomics data using FragPipe-analyst. J Proteome Res.

[34] Gummadi S, Chitti SV, Kang T, Shahi S, Mathivanan S, Fonseka P (2025). ExoCarta 2024: a web-based repository of small extracellular vesicles cargo. J Mol Biol.

[35] Liang X, Ding Y, Zhang Y, Tse HF, Lian Q (2014). Paracrine mechanisms of mesenchymal stem cell-based therapy: current status and perspectives. Cell Transplant.

[36] Viswanathan S, Shi Y, Galipeau J (2019). Mesenchymal stem versus stromal cells: International Society for Cell & Gene Therapy (ISCT®) Mesenchymal Stromal Cell committee position statement on nomenclature. Cytotherapy.

[37] Dominici M, Le Blanc K, Mueller I (2006). Minimal criteria for defining multipotent mesenchymal stromal cells. The International Society for Cellular Therapy position statement. Cytotherapy.

[38] Shin S, Lee J, Kwon Y (2021). Comparative proteomic analysis of the mesenchymal stem cells secretome from adipose, bone marrow, placenta and Wharton’s jelly. Int J Mol Sci.

[39] Braga CL, da Silva LR, Santos RT (2022). Proteomics profile of mesenchymal stromal cells and extracellular vesicles in normoxic and hypoxic conditions. Cytotherapy.

[40] Hanada T, Yoshimura A (2002). Regulation of cytokine signaling and inflammation. Cytokine Growth Factor Rev.

[41] McCormick SM, Heller NM (2015). Commentary: IL-4 and IL-13 receptors and signaling. Cytokine.

[42] Mathern DR, Heeger PS (2015). Molecules great and small: the complement system. Clin J Am Soc Nephrol.

[43] Reichhardt MP, Meri S (2018). Intracellular complement activation - an alarm raising mechanism?. Semin Immunol.

[44] Levy S, Todd SC, Maecker HT (1998). CD81 (TAPA-1): a molecule involved in signal transduction and cell adhesion in the immune system. Annu Rev Immunol.

[45] Fan Y, Pionneau C, Cocozza F (2023). Differential proteomics argues against a general role for CD9, CD81 or CD63 in the sorting of proteins into extracellular vesicles. J Extracell Vesicles.

[46] Stepkowski SM, Chen W, Ross JA, Nagy ZS, Kirken RA (2008). STAT3: an important regulator of multiple cytokine functions. Transplantation.

[47] Kalinski P (2012). Regulation of immune responses by prostaglandin E2. J Immunol.

[48] Kulesza A, Paczek L, Burdzinska A (2023). The Role of COX-2 and PGE2 in the regulation of immunomodulation and other functions of mesenchymal stromal cells. Biomedicines.

[49] Bui TM, Wiesolek HL, Sumagin R (2020). ICAM-1: a master regulator of cellular responses in inflammation, injury resolution, and tumorigenesis. J Leukoc Biol.

[50] Wei D, Tian X, Zhu L, Wang H, Sun C (2023). USP14 governs CYP2E1 to promote nonalcoholic fatty liver disease through deubiquitination and stabilization of HSP90AA1. Cell Death Dis.

[51] Choi S, Park KA, Lee HJ (2005). Expression of Cu/Zn SOD protein is suppressed in hsp 70.1 knockout mice. J Biochem Mol Biol.

[52] Johnston AD, Ebert PR (2012). The redox system in *C. elegans*, a phylogenetic approach. J Toxicol.

[53] Yang HC, Wu YH, Liu HY, Stern A, Chiu DT (2016). What has passed is prolog: new cellular and physiological roles of G6PD. Free Radic Res.

[54] Chen PH, Tjong WY, Yang HC, Liu HY, Stern A, Chiu DT (2022). Glucose-6-phosphate dehydrogenase, redox homeostasis and embryogenesis. Int J Mol Sci.

[55] Park DJ, Duggan E, Ho K (2022). Serpin-loaded extracellular vesicles promote tissue repair in a mouse model of impaired wound healing. J Nanobiotechnology.

[56] Koga Y, Pelizzola M, Cheng E (2009). Genome-wide screen of promoter methylation identifies novel markers in melanoma. Genome Res.

[57] Watt FM (2002). Role of integrins in regulating epidermal adhesion, growth and differentiation. EMBO J.

[58] Chou CW, Huang YK, Kuo TT, Liu JP, Sher YP (2020). An overview of ADAM9: structure, activation, and regulation in human diseases. Int J Mol Sci.

[59] Calzada MJ, Sipes JM, Krutzsch HC (2003). Recognition of the N-terminal modules of thrombospondin-1 and thrombospondin-2 by alpha6beta1 integrin. J Biol Chem.

[60] Bornstein P (2009). Thrombospondins function as regulators of angiogenesis. J Cell Commun Signal.

[61] Tombran-Tink J (2005). The neuroprotective and angiogenesis inhibitory serpin, PEDF: new insights into phylogeny, function, and signaling. Front Biosci.

[62] Acoba MG, Alpergin ESS, Renuse S (2021). The mitochondrial carrier SFXN1 is critical for complex III integrity and cellular metabolism. Cell Rep.

[63] Banerjee M, Joshi S, Zhang J (2017). Cellubrevin/vesicle-associated membrane protein-3-mediated endocytosis and trafficking regulate platelet functions. Blood.

[64] Adams RH, Wilkinson GA, Weiss C (1999). Roles of ephrinB ligands and EphB receptors in cardiovascular development: demarcation of arterial/venous domains, vascular morphogenesis, and sprouting angiogenesis. Genes Dev.

