# Supplementary material for: Proteomic analysis of equine amniotic mesenchymal stromal cells and their extracellular vesicles: comparing their regenerative properties
Source: Extracell Vesicles Circ Nucl Acids. 2026 Mar 9;7(1):302–27. doi: 10.20517/evcna.2025.66 (PMC13074302; doi:10.20517/evcna.2025.66)
Supplement: Supplementary file 1 [file evcna-7-1-302-SupplementaryMaterials.zip › evcna6066-SupplementaryMaterials/Supplementary Figures 1-5.pdf]

Supplementary Materials

Proteomic analysis of equine amniotic mesenchymal stromal cells and their extracellular vesicles: comparing their regenerative properties

Giulia Gaspari[1,#], Alessio Soggiu[2,#], Paola Gagni[3], Pietro Riccaboni[1], Andrea Cappelleri[1], Fausto Cremonesi[1], Anna Lange-Consiglio[1]

[1]Laboratory of Reproduction and Regenerative Medicine, Department of Veterinary Medicine and Animal Science (DIVAS), Università degli Studi di Milano, Lodi 26900, Italy.

[2]Dipartimento di Scienze Biomediche, Chirurgiche e Odontoiatriche, Università degli Studi di Milano, Milan 20133, Italy.

[3]Istituto di Scienze e Tecnologie Chimiche "Giulio Natta" (SCITEC), Consiglio Nazionale delle Ricerche (CNR), Milan 20133, Italy.

[#]Authors contributed equally.

Correspondence to: Prof. Anna Lange-Consiglio, Laboratory of Reproduction and Regenerative Medicine, Department of Veterinary Medicine and Animal Science (DIVAS), Università degli Studi di Milano, Lodi 26900, Italy. E-mail: anna.langeconsiglio@unimi.it

ORCID: Anna Lange-Consiglio (0000-0002-7748-3413)

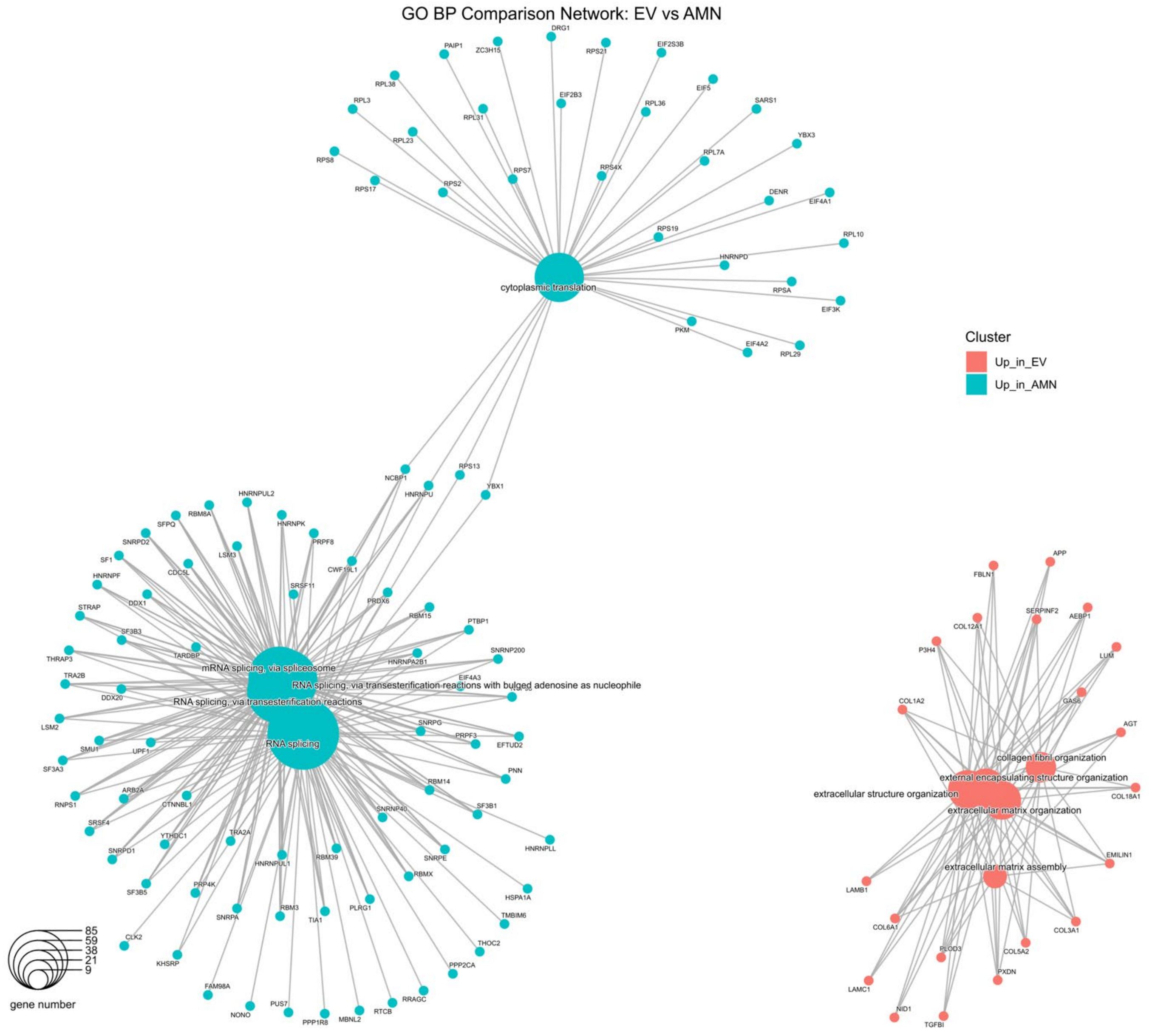

Supplementary Figure 1. Network comparison between eAMC and EV upregulated proteins, based on GO enriched terms for biological processes.

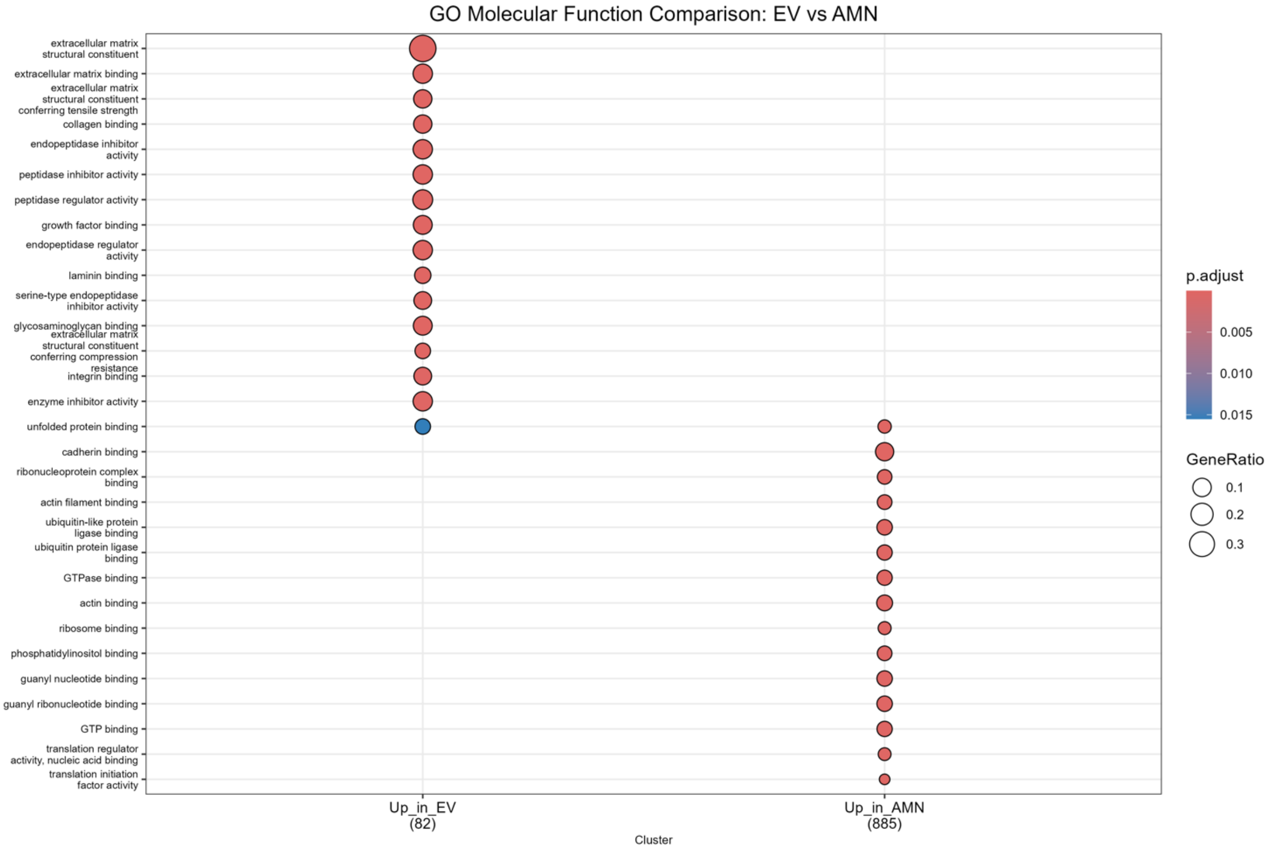

Supplementary Figure 2. Comparison of Gene Ontology Molecular Function significantly enriched terms between eAMCs and EVs.

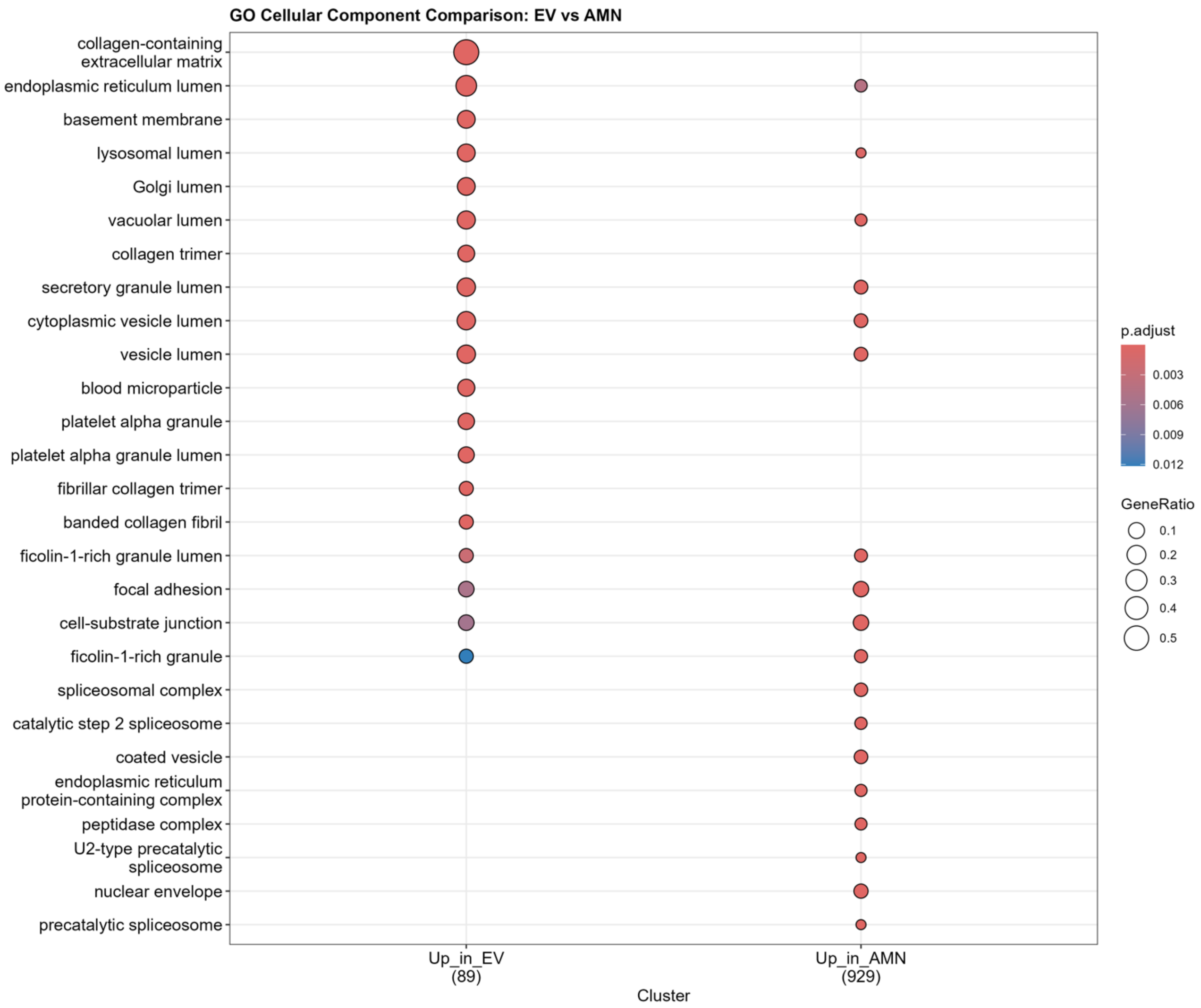

Supplementary Figure 3. Comparison of Gene Ontology Cellular Component significantly enriched terms between eAMCs and EVs.

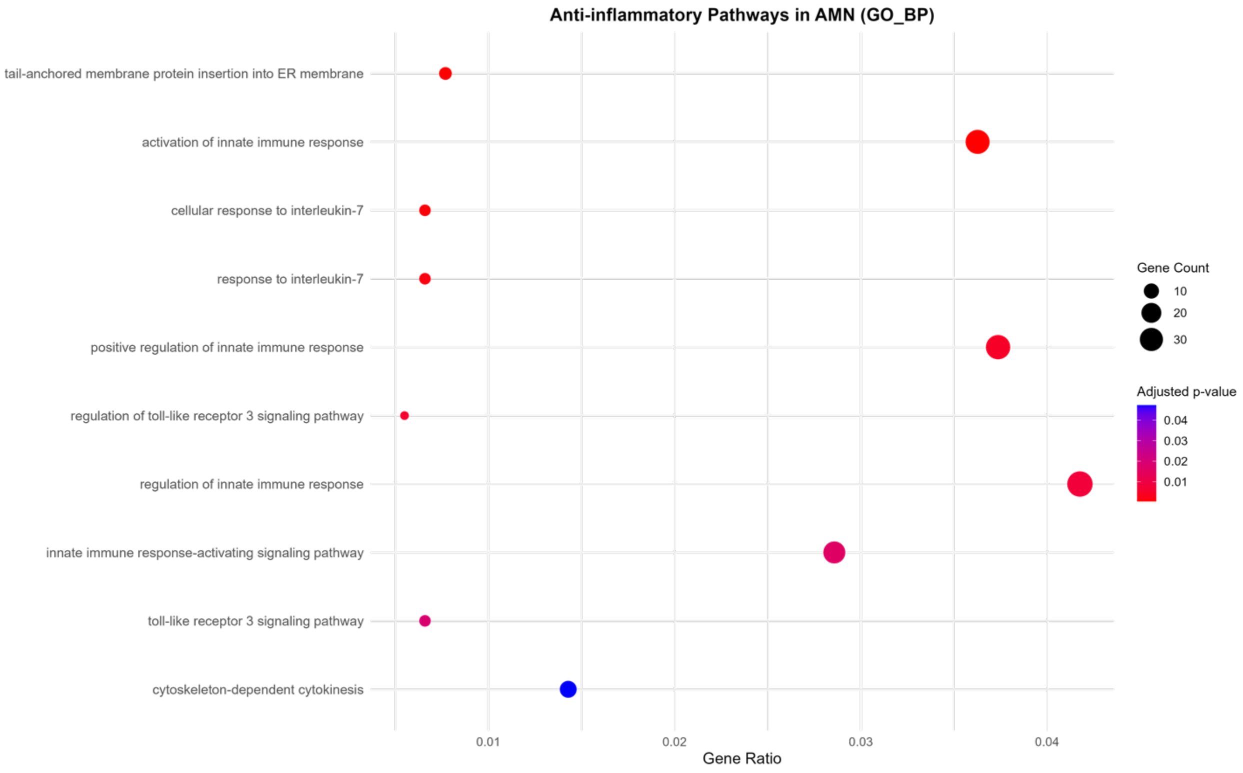

Supplementary Figure 4. GO BP enrichment analysis for anti-inflammatory pathways in eAMC upregulated proteins.

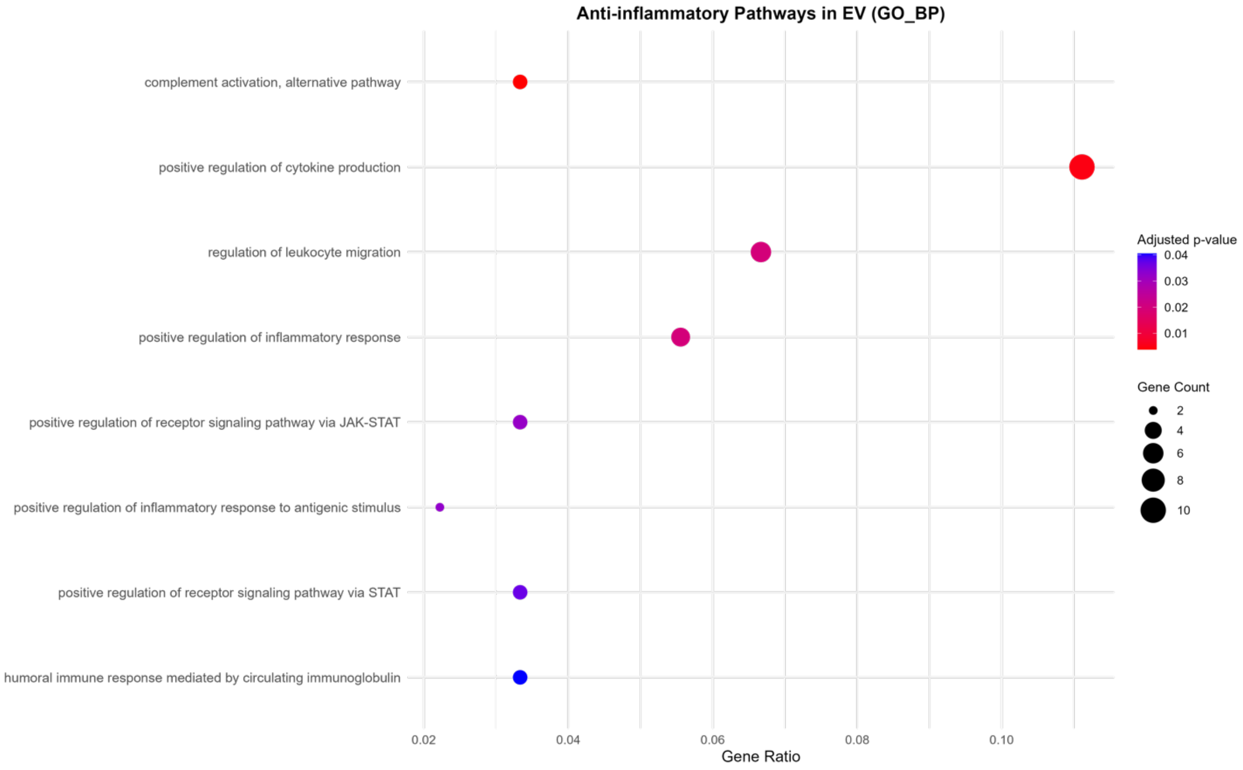

Supplementary Figure 5. GO BP enrichment analysis for anti-inflammatory pathways in EVs upregulated proteins.
